# Using bio-orthogonally catalyzed lethality strategy to generate mitochondria-targeting anti-tumor metallodrugs *in vitro* and *in vivo*

**DOI:** 10.1093/nsr/nwaa286

**Published:** 2020-11-25

**Authors:** Xuling Xue, Chenggen Qian, Qin Tao, Yuanxin Dai, Mengdi Lv, Jingwen Dong, Zhi Su, Yong Qian, Jing Zhao, Hong-Ke Liu, Zijian Guo

**Affiliations:** College of Chemistry and Materials Science, Jiangsu Key Laboratory of Biofunctional Materials, Nanjing Normal University, Nanjing 210023, China; School of Pharmacy, China Pharmaceutical University, Nanjing 210009, China; College of Chemistry and Materials Science, Jiangsu Key Laboratory of Biofunctional Materials, Nanjing Normal University, Nanjing 210023, China; School of Pharmacy, China Pharmaceutical University, Nanjing 210009, China; College of Chemistry and Materials Science, Jiangsu Key Laboratory of Biofunctional Materials, Nanjing Normal University, Nanjing 210023, China; School of Pharmacy, China Pharmaceutical University, Nanjing 210009, China; College of Chemistry and Materials Science, Jiangsu Key Laboratory of Biofunctional Materials, Nanjing Normal University, Nanjing 210023, China; College of Chemistry and Materials Science, Jiangsu Key Laboratory of Biofunctional Materials, Nanjing Normal University, Nanjing 210023, China; State Key Laboratory of Coordination Chemistry, Chemistry and Biomedicine Innovation Center, School of Chemistry and Chemical Engineering, Nanjing University, Nanjing 210023, China; College of Chemistry and Materials Science, Jiangsu Key Laboratory of Biofunctional Materials, Nanjing Normal University, Nanjing 210023, China; State Key Laboratory of Coordination Chemistry, Chemistry and Biomedicine Innovation Center, School of Chemistry and Chemical Engineering, Nanjing University, Nanjing 210023, China

**Keywords:** metallodrug, cancer therapy, mitochondrial targeting, bio-orthogonally catalyzed lethality, bio-orthogonal reaction

## Abstract

Synthetic lethality was proposed nearly a century ago by geneticists and recently applied to develop precision anti-cancer therapies. To exploit the synthetic lethality concept in the design of chemical anti-cancer agents, we developed a bio-orthogonally catalyzed lethality (BCL) strategy to generate targeting anti-tumor metallodrugs both *in vitro* and *in vivo.* Metallodrug Ru-rhein was generated from two non-toxic species Ru-N_3_ and rhein-alkyne via exclusive endogenous copper-catalyzed azide alkyne cycloaddition (CuAAC) reaction without the need of an external copper catalyst. The non-toxic species Ru-arene complex Ru-N_3_ and rhein-alkyne were designed to perform this strategy, and the mitochondrial targeting product Ru-rhein was generated in high yield (>83%) and showed high anti-tumor efficacy *in vitro*. This BCL strategy achieved a remarkable tumor suppression effect on the tumor-bearing mice models. It is interesting that the combination of metal-arene complexes with rhein via CuAAC reaction could transform two non-toxic species into a targeting anti-cancer metallodrug both *in vitro* and *in vivo*, while the product Ru-rhein was non-toxic towards normal cells. This is the first example that exclusive endogenous copper was used to generate metal-based anti-cancer drugs for cancer treatment. The anti-cancer mechanism of Ru-rhein was studied and autophagy was induced by increased reactive oxygen species and mitochondrial damage. The generality of this BCL strategy was also studied and it could be extended to other metal complexes such as Os-arene and Ir-arene complexes. Compared with the traditional methods for cancer treatment, this work presented a new approach to generating targeting metallodrugs *in vivo* via the BCL strategy from non-toxic species in metal-based chemotherapy.

## Teaser

This work established a 'bioorthogonally catalyzed lethality' strategy to generate tumor-targeting metallodrugs both in vitro and in vivo.

## INTRODUCTION

Synthetic lethality, named by Theodore Dobzhansky in 1946, was an interesting genetic phenomenon originally discovered in *Drosophila melanogaster*: loss of function of one of two genes alone has little effect on *Drosophila* viability, whereas inactivation of both genes simultaneously leads to *Drosophila* death [1]. Synthetic lethality was predicted to be used for the development of anti-cancer drugs [2,3], and a series of poly ADP-ribose polymerase (PARP) inhibitors such as Olaparib (2014), Rucaparib (2016) and Niraparib (2017), have received FDA approval for use in clinical cancer therapies, verifying that synthetic lethality has provided interesting information for the development and clinical approval of other therapies [4]. The advantage of the synthetic lethality-based approach is that it adds a degree of cancer targeting. For example, PARP inhibitors can pose specific cytotoxicity only in cancer cells with a breast cancer (BRCA) gene mutation, no cytotoxicity to the normal cells that do not carry the mutation [5]. However, there are a large number of genetic mutations in cancer cells; it is complicated, time-consuming and expensive to simultaneously screen for two specific genes that can combine and cause lethality.

Inspired by the synthetic lethality feature of *Drosophila*, a bio-orthogonally catalyzed lethality (BCL) strategy could be a promising way to avoid screening for specific genes and selectively kill cancer cells while minimizing the side effects towards normal cells. Screening two non-toxic chemical entities that only react efficiently in cancer cells but not normal cells to produce toxic products would make it easier to achieve the synthetic lethality. Bio-orthogonal chemistry has now become one of the most powerful tools in drug discovery and chemical biology [6–9]. Bio-orthogonal chemistry can specifically label and probe a wide variety of biomolecules in living cells and animals [10–13], and is used for drug design and synthesis *in situ* [14–17]. It refers to the chemical reactions that can be performed in living cells or tissues without interfering with the biochemical reaction of the organism itself. The copper-catalyzed azide-alkyne cycloaddition reaction (CuAAC) is the most common type of bio-orthogonal reaction, most of which use copper as the catalyst [18,19]. The catalyst Cu species plays a key role in the life system and frequently serves as a catalytic cofactor for enzymes that function in antioxidant defense, iron homeostasis, cellular respiration and a variety of biochemical processes [20]. However, the copper concentrations were reported to be significantly elevated in cancerous tissues compared to normal tissues [21,22], which rendered the copper ion as a target for cancer treatment [23,24]. Therefore, taking advantage of the higher Cu levels in tumor cells to generate cancer-specific drugs *in situ* via CuAAC will help achieve targeting treatment towards tumor cells and avoid toxicity to normal cells. To date, the traditional chemotherapy treatments for cancer include at least two steps: synthesis and purification of certain anti-cancer drugs from raw materials through a series of organic syntheses and purification, and then injection or oral administration into the human body for treatment. It will be a revolutionary breakthrough if the targeting anti-cancer drugs could be generated precisely in live cancer cells and animals from the non-toxic materials in the tumor microenvironment.

**Figure 1.**
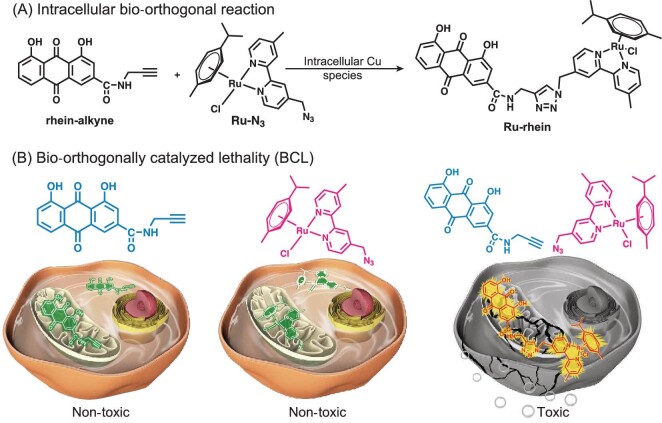
(A) By utilizing the high efficiency and selectivity of the bio-orthogonal reaction, the natural product derivative rhein-alkyne and ruthenium precursor Ru-N_3_ were designed to generate the anti-cancer metallodrug Ru-rhein *in situ* with copper species as the catalyst. Besides, the higher copper levels in tumor cells might help realize the bio-orthogonal reaction and kill the tumor cells selectively without the need of an external copper catalyst. (B) Bio-orthogonally catalyzed lethality (BCL) between rhein-alkyne and Ru-N_3_ in tumor cells. Both rhein-alkyne and Ru-N_3_ exhibited poor or no cytotoxic activities; once they encountered each other inside the cancer cells, the *in situ* cycloaddition product Ru-rhein via bio-orthogonal reaction exhibited high cytotoxicity against cancer cells, realizing BCL.

Metallodrugs such as ruthenium-arene (Ru-arene) complexes have aroused considerable interest as the basis for new anti-cancer agents due to their specificity in cancer cells, high water solubility, clearance properties and low side effects, which represent desirable characteristics for ideal anti-cancer drugs [25–33]. Results show that the potential target of Ru-arene complexes in the cell might be DNA and their anti-cancer mechanism is quite different from that of cisplatin [26,28–31]. The natural product rhein has been used as a laxative and stomach drug, exhibiting low toxicity towards normal cells [34,35] and mainly located in mitochondria, which may be helpful to increase the mitochondria accumulation of metal species and ultimately improve the anti-tumor activity of the metal complex through mitochondrial targeting if used as ligand. Herein, we describe a BCL strategy to generate metallodrugs in cancer cells *in situ* via CuAAC between two non-toxic compounds, Ru-arene complex Ru-N_3_ and mitochondria-targeting rhein-alkyne (Fig. 1, Scheme S1). The CuAAC reaction mechanism, determination of the BCL product and its yield *in vitro* were studied by electrospray ionization mass spectrometry (ESI-MS) and ICP-MS (inductively coupled plasma-mass spectrometry); the mitochondrial-targeting ability, anti-tumor cytotoxicity and anti-cancer mechanism were studied by state-of-the-art chemical and physical techniques and cell biological assays. The generality of the BCL strategy was evaluated by using other metal complexes such as Os-arene and Ir-arene complexes. The application of BCL *in vivo* was also performed in the xenograft A549 tumor-bearing mice model.

## RESULTS AND DISCUSSION

### Design of the BCL agents

Compound rhein-alkyne was synthesized by modifying rhein with mono-propargylamine through the amidation reaction, and Ru-N_3_ was obtained by the reaction of dichloro(*p*-cymene) ruthenium dimer with 2,2′-dipyridyl-N_3_ (Scheme S1). For the BCL strategy, all the experiments, such as the mitochondrial-targeting ability, anti-tumor cytotoxicity, reaction mechanism and determination, and anti-cancer mechanistic studies of BCL product *in vitro* were carried out by incubation of the 1 : 1 mixture of rhein-alkyne and Ru-N_3_ with a range of cancer cell lines or normal cells, respectively, and the application of BCL *in vivo* was performed in the xenograft A549 tumor-bearing mice model by injection of the 1 : 1 mixture of rhein-alkyne and Ru-N_3_. All the comparative experiments were repeated by using chemically synthesized product Ru-rhein and the same procedures. The comparison reactions between the 1 : 1 rhein-alkyne and Ru-N_3_ in the chemical reactor were performed with or without additional Cu catalysts, respectively. Only the reaction with additional Cu catalysts gave the final product Ru-rhein, which was purified and characterized by ^1^H nuclear magnetic resonance (NMR) and ESI-MS spectroscopy (Figs S1–S5). Besides, the UV-vis and photoluminescence spectra of rhein-alkyne and Ru-rhein (10 μM) exhibited that both of them showed good absorption and emission properties (λ_abs_ = ∼430 nm, λ_em_ = ∼600 nm, Fig. S6), which favored monitoring their cell uptake and intracellular distribution by confocal fluorescence imaging. Ru-rhein is represented as the BCL product Ru-rhein generated *in vitro* or *in vivo*, and the chemical synthesized product is defined as chemically synthesized Ru-rhein *infra.*

### Mitochondrial-targeting of the CuAAC product

**Figure 2.**
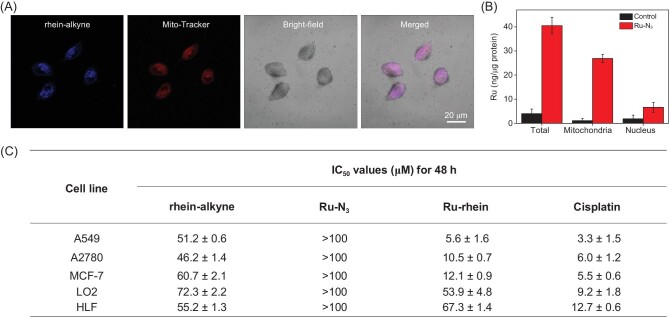
The successful cycloaddition reaction between rhein-alkyne and Ru-N_3_ in A549 cells. (A) Confocal microscopy images of A549 cells incubated with rhein-alkyne (10 μM) for 12 h, then co-localized with Mito-Tracker Deep Red. The Pearson correlation coefficients were 0.90, respectively. λ_ex_ = 405 nm, λ_em_ = 520–700 nm for rhein-alkyne; λ_ex_ = 543 nm, λ_em _= 580–700 nm for Mito-Tracker. Scale bar: 20 μm. (B) Cellular uptake of Ru in A549 cells with 10 μM Ru-N_3_ determined by ICP-MS (ng/μg protein), verifying the co-localization of rhein-alkyne and Ru-N_3_ in the mitochondria. (C) The IC_50_ (μM) values for rhein-alkyne, Ru-N_3_, Ru-rhein and cisplatin against cancer and normal (HLF, LO2) cell lines for 48 h, indicating that Ru-rhein exhibited enhanced cytotoxicity towards cancer cells and low toxicity to normal cells, confirming successful *in situ* BCL in cancer cells.

To verify the possibility of BCL via CuAAC in living cells, the intracellular distribution of rhein-alkyne and Ru-N_3_ was conducted using confocal microscopy and ICP-MS methods. We have detected the enrichment of Ru-N_3_ and rhein-alkyne in different organelles after a short time of incubation. After incubation of 10 μM rhein-alkyne for 4 h or 12 h, the blue fluorescence of rhein-alkyne and red fluorescence of Mito-Tracker overlapped well, with a Pearson's correlation coefficient of 0.89 (Fig. S7) or 0.90 (Fig. 2A). The intracellular amount of Ru was very small after incubation of 10 μM Ru-N_3_ for 4 h, but after 8 or 12 h incubation Ru-N_3_ could enter cells in large amounts and ∼65% or 66% were enriched in mitochondria (Fig. 2B and Fig. S8). These suggested that both rhein-alkyne and Ru-N_3_ were mainly accumulated in the mitochondria of the cells after 8 to 12 h incubation. Moreover, the cellular location of the chemically synthesized Ru-rhein in A549 cells also indicated that the cycloaddition product Ru-rhein was enriched in the mitochondria (Fig. S9). All these results could serve as a basis for the *in situ* azide-alkyne cycloaddition between rhein-alkyne and Ru-N_3_.

### Anti-tumor cytotoxicity of BCL product *in vitro*

We further determined the anti-tumor cytotoxicities of rhein-alkyne, Ru-N_3_, mixture of rhein-alkyne and Ru-N_3_ (molar ratio of 1 : 1) towards a range of cancer cell lines or normal LO2 and HLF cells through the 3-(4,5)-dimethylthiahiazo (-z-y1)-3,5-di-phenytetrazoliumromide (MTT) assays, with cisplatin as the control group. As shown in Fig. 2C, both rhein-alkyne and Ru-N_3_ exhibited little or no cytotoxicity towards cancer cells and two normal cell lines, with the IC_50_ values ranging from ∼46.2 to over ∼100.0 μM. However, the growth of the cancer cells was significantly inhibited and the IC_50_ value was 5.6 μM, as toxic as that of cisplatin (3.3 μM), when 1 : 1 mixture of rhein-alkyne and Ru-N_3_ was added to the A549 cell culture (Fig. S10). Furthermore, the 1 : 1 mixture of rhein-alkyne and Ru-N_3_ also exhibited high cytotoxicity against A2780 and MCF-7 cells, with the IC_50_ values of 10.5 μM and 12.1 μM, respectively. More importantly, the mixture of rhein-alkyne and Ru-N_3_ exhibited low toxicity against normal LO2 and HLF cells with IC_50_ values of ∼53.9 and ∼67.3 μM (Fig. S11), much lower than that of cisplatin, indicating high selectivity towards cancer cells. These results suggested that the BCL strategy was realized and the anti-tumor efficacy was activated when cycloaddition reaction between rhein-alkyne and Ru-N_3_ occurred in cancer cells *in situ*.

### Investigation on the BCL product and CuAAC reaction mechanism *in vitro*

Based on the high anti-tumor activity of 1 : 1 mixture of rhein-alkyne and Ru-N_3_ in cancer cell lines, we hypothesized a metallodrug with high efficacy was generated in the cancer cells. Several experiments were carried out to determine the BCL product *in vitro.* After the A549 cells were treated with 1 : 1 mixture of rhein-alkyne and Ru-N_3_, Ru-N_3_ or rhein-alkyne alone, as well as chemically synthesized Ru-rhein, respectively, the main ingredient inside the cancer cells was characterized by using ESI-MS techniques. For

comparison, complexes Ru-N_3_ or rhein-alkyne, and the chemically synthesized Ru-rhein were also characterized by ESI-MS techniques in methanol solution, respectively. As shown in Figs S12 and S13, it is interesting to note that after the non-toxic species Ru-N_3_ or rhein-alkyne enters into the A549 cells, their composition remains unchanged, for the positive-ion MS peak (m/Z) at 322.33 assigned for rhein-alkyne or the peak at 496.08 for [Ru-N_3_ − PF_6_]^+^; those results match well with the samples measured in methanol solutions. Only one positive-ion peak (m/Z) at 817.25 was observed for the chemically synthesized Ru-rhein in methanol solution (Fig. S5), which was assignable to the cycloaddition product [Ru-rhein - PF_6_]^+^ (817.15). It is remarkable that the same two positive-ion peaks (m/Z) at 817.25 and 909.17 were observed for the A549 cells treated with the 1 : 1 mixture of rhein-alkyne and Ru-N_3_ (Fig. 2A) and for the chemically synthesized Ru-rhein (Fig. 2B), respectively, and that these isotope patterns for peaks at 817.25 were the same as that observed for the chemically synthesized Ru-rhein in methanol solution, indicating the formation of cycloaddition product Ru-rhein when rhein-alkyne and Ru-N_3_ encountered each other in A549 cells. The ESI-MS peak at 909.17 might be attributed to the signal of its lysine adduct for [Ru-rhein − PF_6_ − Cl − H^+^ + lysine]^+^, which was calculated m/Z of 909.34. These results confirmed the successful generation of Ru-rhein *in vitro* via alkyne-azide cycloaddition of rhein-alkyne and Ru-N_3_ without additional Cu catalysts in living cancer cells.

It is worth noting that, without a copper catalyst (CuI or CuSO_4_/NaAsc), the alkyne-azide cycloaddition reaction could not react between Ru-N_3_ and rhein-alkyne in N,N-dimethylformamide (DMF) solution, even under the severe conditions of a long time and high temperature. However, such azide-alkyne cycloaddition reaction could react easily without a copper catalyst within the intracellular compartment *in situ* and exhibited high reaction yield for Ru-rhein in mild conditions, which might be attributed to the higher levels of endogenous copper in tumor cells. It is reported that the content of copper ions in lung cancer patients is 1.3–2.0 times more than that of non-cancerous patients. Scanni [36] found that the copper concentration of normal patients was ∼143.03 μg/dL, while the copper levels in cancer patients increased to ∼188 ug, about 1.3 times higher. Díez [37] also pointed out that the copper ion level of Stage III cancer patients, being ∼150 μg/dL and 100 μg/dL, was 1.5 times more than that of normal patients. Thus, we further detected the concentrations of copper species in several cancer and normal cell lines. As shown in Fig. S14, the copper concentrations are ∼41.3, 67.9, 55.1, 24.2 and 21.6 ppb/10^7^ cells in A2780, A549, MCF-7, LO2 and HLF cells respectively, proving that the copper concentrations were indeed higher in the cancer cells than in the normal cells. This was consistent with the cytotoxicities of Ru-rhein, again verifying our design that the higher levels of copper species could enhance the selectivity against cancer cells and the higher levels of intracellular copper species resulted in higher cytotoxicity of Ru-rhein (Table S1). Overall, this suggested that the elevated copper levels in cancer cells might be an important copper source for tumor cells to achieve BCL.

### Yields of BCL product Ru-rhein *in vitro*

**Figure 3.**
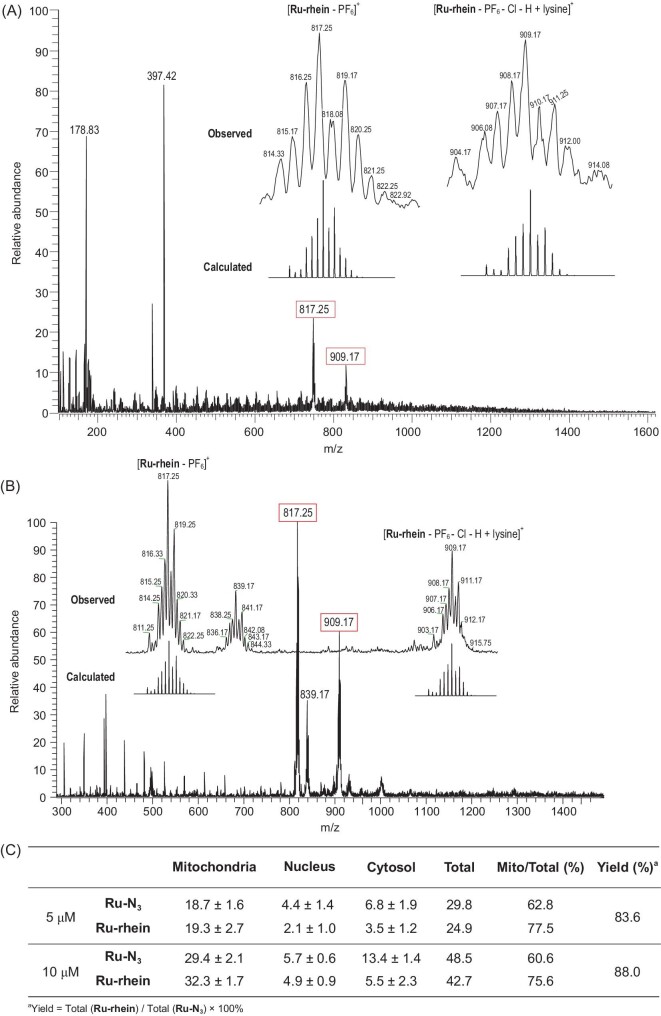
ESI-MS results of A549 cells after treatment with 10 μM Ru-rhein without Cu(I) catalysts (A) and 10 μM chemically synthesized Ru-rhein (B) for 12 h. The m/Z peak 817.25 is assigned as [Ru-rhein − PF_6_]^+^ (calculated 817.29). The m/Z peak 909.17 is assigned as [Ru-rhein − Cl − PF_6_ − H + lysine]^+^ (calculated 909.34). The similar m/Z peaks at 817.25 of (A) and (B) indicated the successful *in situ* CuAAC between Ru-N_3_ and rhein-alkyne in A549 cells without additional Cu(I) catalysts. (C) Cellular uptake of Ru in A549 cells with 5 and 10 μM Ru-N_3_ or Ru-rhein incubation determined by ICP-MS (ng/μg protein).

The ICP-MS technique was used for the first time to determine the yields of BCL product Ru-rhein *in vitro*. Two concentrations (5 and 10 μM) of Ru-N_3_ or 1 : 1 mixture of Ru-N_3_ and rhein-alkyne were incubated with A549 cells for 24 h and monitored by ICP-MS techniques. As shown in Fig. 3C, the Ru content in the mitochondria of 5 μM Ru-N_3_-loaded A549 cells was determined to be ∼18.7 ng/μg protein, accounting for 62.8% of the total Ru content. When the A549 cells were treated with 5 μM 1 : 1 mixture of Ru-N_3_ and rhein-alkyne, the Ru content in the mitochondria reached ∼19.3 ng/μg protein, accounting for 77.5% of the total quantity, implying that introduction of rhein into the metallodrug could improve the mitochondrial enrichment of Ru species. The yield of Ru-rhein *in vitro* was up to ∼83.6%, which was obtained via dividing the total content of Ru-rhein in whole A549 cells (24.9 ng) by the total content of Ru-N_3_ in whole A549 cells (29.8 ng) × 100%. Similarly, when the A549 cells were incubated with 10 μM Ru-N_3_ or 1 : 1 mixture of Ru-N_3_ and rhein-alkyne, respectively, the percentage of Ru in the mitochondria increased from 60.6% to 75.6%, and the yield of Ru-rhein *in vitro* was ∼88.0%. These results indicated that the existence of rhein could enhance the targeting mitochondrial of Ru species, and a higher concentration of 1 : 1 mixture of Ru-N_3_ and rhein-alkyne help to generate product in higher yield, both contributing to a greatly improved BCL efficiency.

### Mechanistic studies of cell death by BCL product Ru-rhein *in vitro*

Several cell biological assays were used to investigate the mechanism of cell death induced by Ru-rhein *in vitro*. Since the Ru-rhein mainly localized in mitochondria, we supposed that cell death might be related to mitochondrial death pathways. Mitochondria are a major source and may act as a point of integration for death signals originating from both the extrinsic and intrinsic pathways [38], such as an elevation of reactive oxygen species (ROS) levels, variations in mitochondrial membrane potential and the generation of autophagy. The impact of Ru-rhein on intracellular ROS level, mitochondrial membrane potential and autophagy-related protein expression was investigated. When the A549 cells were treated with Ru-rhein, the levels of intracellular ROS increased and the mitochondrial membrane potential was disturbed severely in a concentration-dependent manner (Figs S15–S17). The excess ROS caused obvious oxidative stress and mitochondria dysfunction in A549 cells exposed to Ru-rhein, and we explored whether the cytotoxic effect of Ru-rhein was related to the induction of autophagy.

**Figure 4.**
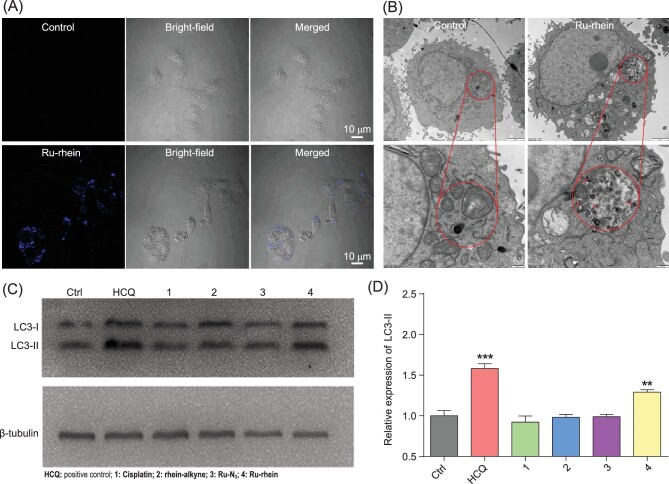
The cell death mechanism after treatment with Ru-rhein. (A) A549 cells were stained with 5 μM monodansylcadaverine (MDC) for 20 min after treatment with Ru-rhein (5 μM) separately for 24 h and then analyzed by fluorescence microscopy. The increased fluorescence indicated the Ru-rhein-induced formation of autophagosomes. λ_ex_ = 405 nm, λ_em _= 430–600 nm for MDC. Scale bar: 10 μm. (B) Transmission electron microscopy imaging showed numerous autophagic vacuoles (red arrows) in A549 cells after treatment with 5 μM Ru-rhein for 24 h. (C and D) The autophagy-related protein expression of LC3 induced by 5 μM cisplatin (1), rhein-alkyne (2), Ru-N_3_ (3) and Ru-rhein (4), respectively, in A549 cells for 24 h, observed by western blot analysis. HCQ treatment as the positive control induced autophagy. The data represent the mean ± SEM of three different experiments. ^**^P < 0.01, ^***^P < 0.001. These results indicated that Ru-rhein mainly caused autophagic cell death of A549 cells.

We further examined the autophagy by using confocal laser scanning microscopy, transmission electron microscopy (TEM) and western blot analysis. Monodansylcadaverine (MDC) staining is a specific assay for autolysosomes, which is dependent on a second ubiquitin-like protein-binding system (located on the membrane of the autophagy vesicle, which can be specifically bound to it) [39]. As shown in Fig. 4A, positively dot-like structures were observed both in the peripheral region of the nucleus and selectively aggregated in the autophagy vesicles when A549 cells were treated with Ru-rhein for 12 h, while no blue fluorescence was observed in the cytoplasm in the control group without Ru-rhein incubation, indicating that Ru-rhein induced the formation of autophagosomes. Ru-rhein-induced autophagy was also monitored by TEM (Fig. 4B). Numerous double-membraned cytosolic autophagic vacuoles in A549 cells were observed after treatment with Ru-rhein for 24 h, while such autophagic vacuoles were not found in the control group without drug treatment. These results verified that significant autophagy occurred in A549 cells after treatment with Ru-rhein.

**Figure 5. fig5:**
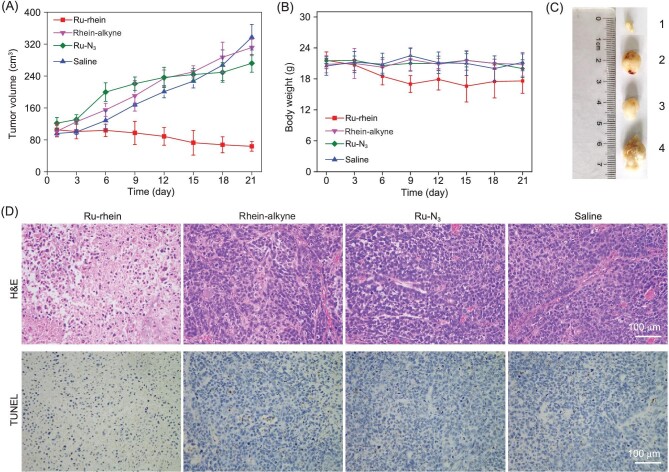
The successful BCL *in vivo*. Nude mice bearing A549 tumors were intratumorally injected with various samples at a dosage of 8.0 mg/kg body weight once every three days. The samples included Ru-rhein, rhein-alkyne, Ru-N_3_ and saline. (A) Changes in tumor volume during 18 days of the indicated treatments. Data are the mean ± SD (n = 6). (B) Changes in mice weight during 18 days of the indicated treatments. Data are the mean ± SD (n = 6). (C) Photographs of tumors of each group obtained on the 18th day of treatment. 1: Ru-rhein; 2: rhein-alkyne; 3: Ru-N_3_; 4: Saline. (D) Detection of cell death in the tumor tissues after treatment with H&E and TUNEL staining assay after treatment with different samples. Scale bars are 100 μm.

Moreover, microtubule-associated protein light chain 3 (LC3), as a mammalian homolog of yeast Atg8, has been used as a specific marker to detect autophagy; occurrence of autophagy will render LC3 conjugate to phosphatidylethanolamine and target to autophagic membranes [40]. Western blotting experiments were then performed using an anti-LC3 antibody to verify the expression of related

autophagy proteins for A549 cells treated with Ru-rhein. After treatment with cisplatin, rhein-alkyne and Ru-N_3_ for 24_ _h, the expression of LC3-II in A549 cells was almost negligible compared to that in the positive control hydroxychloroquine (HCQ) (Fig. 4C). However, an obvious increase in LC3-II protein expression was observed when A549 cells were treated with Ru-rhein, and the ratio of LC3-I to LC3-II expression was 1.3 times higher than that in the control group without drug treatment (Fig. 4D),5 proving the induction of autophagy by Ru-rhein, which was consistent with the results of fluorescence confocal images of MDC and TEM images. Taken together, this evidence indicates that Ru-rhein mainly caused autophagic cell death of A549 cells through alterations in oxidative stress and mitochondria membrane potential. Similarly, the main mechanism of chemically synthesized Ru-rhein in causing cell death was also autophagy induced by increased reactive oxygen species and mitochondrial damage (Fig. S18). These results also indicated that the BCL strategy enabled the formation of mitochondria-targeted anti-tumor metallodrug Ru-rhein *in vitro*. In addition, we also investigated the effects of Ru-rhein treatment on cell cycle and apoptosis of A549 cells, which showed Ru-rhein did not cause cell death by blocking the cell cycle and only caused a small amount of increased population of early apoptotic cells (Figs S19 and S20).

### Application of BCL *in vivo*

Since BCL was achieved successfully in cancer cells *in vitro*, we further investigated whether BCL could be triggered by endogenous Cu in A549 tumor-bearing mice models. The mice were divided randomly into four groups (six mice/group) and intratumorally injected with Ru-rhein (1 : 1 mixture

of Ru-N_3_ and rhein-alkyne), rhein-alkyne, Ru-N_3_ and saline once every three days for 18 days. As shown in Fig. 5A and C, the growth of the tumors was significantly inhibited after the mice were treated with Ru-rhein, while the mice treated with rhein-alkyne, Ru-N_3_ or saline showed rapid growth of the tumors. These results suggested that Ru-rhein displayed high anti-cancer efficacy and achieved BCL *in vivo*, which was consistent with the *in vitro* results. Besides, the body weight of the mice treated with different samples showed no obvious change during the cancer treatment (Fig. 5B), indicating that Ru-rhein displayed little toxicity to the mice.

The haematoxylin and eosin (H&E) staining and terminal deoxynucleotidyl transferase dUTP nick-end labeling (TUNEL) staining assays were further performed to evaluate the efficacy of different treatments (Fig. 5D). The images of H&E-stained tumor tissue exhibited distinct necrosis of tumor cells treated by Ru-rhein, and the images obtained from the TUNEL staining also showed the highest level of cell death in the tumor tissue of the mice treated with Ru-rhein. The images of H&E-stained tumor tissue and TUNEL staining after treatment of the control groups (rhein-alkyne, Ru-N_3_ and saline) could not exhibit obvious tumor necrosis. Besides, the chemically synthesized Ru-rhein could also induce a high level of cell death in the tumor tissue and significantly inhibit the growth of the tumor *in vivo* (Fig. S21). Taken together, these results suggested that Ru-rhein exhibited satisfactory therapeutic effects *in vivo*, proving the BCL strategy can also be applied in living mice.

### Generality of the BCL strategy

To probe the general applicability of BCL, Os-arene and Ir-arene complexes were used to form BCL products via azide-alkyne cycloaddition between two non-toxic species of rhein-alkyne and M-N_3_ (M=Os, Ir) in cancer cells (Scheme S1). The chemically synthesized complexes were characterized by ^1^H NMR and ESI-MS spectroscopy (Figs S22–S27). The anti-proliferative activities of the 1 : 1 mixture of rhein-alkyne and M-N_3_ (M=Os, Ir) against several cell lines were determined (Table S2); both the rhein-alkyne and M-N_3_ (M=Os, Ir) complexes exhibited poor or no cytotoxicity, while the cycloaddition products M-rhein (M=Os, Ir) displayed high cytotoxicities with the IC_50_ value decreasing to 13.3 μM. This not only confirmed the successful cycloaddition reaction between rhein-alkyne and M-N_3_ (M=Os, Ir) species in living cells, but also showed enhanced cytotoxicities towards cancer cells. These results indicated the potential generality of the BCL strategy, and this strategy might be extended to a wider range for developing metallodrugs for cancer chemotherapy.

## CONCLUSIONS

Inspired by synthetic lethality, we selected two non-toxic components which could generate efficient anti-cancer species only in cancer cells *in situ* without an external catalyst. Such strategy is named the BCL strategy. Catalytic products M-rhein (M=Ru, Os or Ir) were generated *in vitro* by adopting an exclusive endogenous CuAAC reaction between M-N_3_ and mitochondria-targeting rhein-alkyne without an additional copper catalyst. This BCL strategy generated targeting metallodrugs with high anti-tumor efficacy both *in vitro* and *in vivo* and could be extended to a wider range for metallodrugs cancer therapy. Moreover, this BCL strategy could produce the remarkable tumor suppression effect on tumor-bearing mice models. The combination of metal-arene complexes with rhein via CuAAC reaction could transform two non-toxic species into targeting anti-cancer metallodrugs M-rhein (M=Ru, Os and Ir), which are non-toxic to normal cells. The autophagy was the main cause for cancer cell death and this was consistent with the fact that Ru-rhein is a mitochondria-targeting anti-tumor metallodrug. The generality of this strategy was also studied by replacing the metal species with non-toxic Os and Ir precursors. This strategy not only avoided complicated pre-synthesis of the metallodrugs but was also expected to overcome the decomposition and inactivation of the pre-synthesized cycloaddition product *in vitro* and *in vivo*. Generation of metallodrugs *in vivo* by the BCL strategy might be a new approach compared with the traditional treatment pattern of anti-cancer metallodrugs, and might provide a new pattern and platform for precise, targeted and efficient anti-cancer chemotherapy, especially for metal-based drugs.

## Supplementary Material

nwaa286_Supplemental_File
