# Supplementary material for: Using bio-orthogonally catalyzed lethality strategy to generate mitochondria-targeting anti-tumor metallodrugs in vitro and in vivo
Source: Natl Sci Rev. 2020 Nov 25;8(9):nwaa286. doi: 10.1093/nsr/nwaa286 (PMC8433091; doi:10.1093/nsr/nwaa286)
Supplement: nwaa286_Supplemental_File [file nwaa286_supplemental_file.docx]

***SI Appendix***

***for***

**Using Bioorthogonally Catalyzed Lethality Strategy** **to Generate Mitochondria-Targeting Antitumor Metallodrugs *in vitro* and *in vivo***

Xuling Xue^1†^, Chenggen Qian^3†^, Qin Tao^1^, Yuanxin Dai^3^, Mengdi Lv^1^, Jingwen Dong^3^, Zhi Su^1^, Yong Qian^1^, Jing Zhao*^2^, Hongke Liu*^1^, and Zijian Guo*^2^

^1^ College of Chemistry and Materials Science, Jiangsu Key Laboratory of Biofunctional Materials, Nanjing Normal University, Nanjing, P. R. China

^2^ State Key Laboratory of Coordination Chemistry, Chemistry and Biomedicine Innovation Center, School of Chemistry and Chemical Engineering, Nanjing University, Nanjing, P. R. China

^3^ School of Pharmacy, China Pharmaceutical University, Nanjing, P. R. China

Email: jingzhao@nju.edu.cn; liuhongke@njnu.edu.cn; zguo@nju.edu.cn.

† Xuling Xue and Chenggen Qian contributed equally to this work.

**Experimental section**

**Materials and instrumentation**

Unless otherwise specified, all the solvents and reagents were purchased from commercial suppliers and used without further purification. The ^1^HNMR data were recorded at 400 MHz on a Bruker DRX-400 NMR spectrometer with tetramethylsilane (Si(CH_3_)_4_) as the internal standard. The ESI-MS data were determined using the LCQ electrospray mass spectrometer (Thermo Finnigan). The confocal imagings were performed on confocal laser scanning microscope (Nikon TI-E-A1R, Japan). TEM results were observed using a transmission electron microscope (H7650, Hitachi, Japan) at 80 kV in high contrast mode. Flow cytometric analysis was obtained on flow cytometer (BD FACSverse, USA). Western blotting experiments were conducted on Mini-Protean Tetra System (BIO RAD, Power PacTM HC, Sinapore) and the signals were enhanced by Tanon High-sig ECL Western Blotting Substrate. The H&E and TUNNEL data were collected on a fluorescence microscope (ECLIPSE Ci-L, Nikon).

**Synthesis of rhein-alkyne**

0.142 g rhein (0.5 mmol), 0.095 g EDCI (0.5 mmol) and 10 µL Et_3_N were dissolved in anhydrous DMF (10 mL) and cooled to 0 ^o^C and stirred at ice bath for 20 min. Then 0.054 g HOBt (0.4 mmol) and 0.061 g DMAP (0.5 mmol) were added to the reaction mixture and stirred at room temperature overnight. After the reaction completed, the DMF solvent was removed under pressure, and the compound was purified by column chromatography using 10% methanol in dichloromethane as eluent and 0.13 g pure product were obtained with the yield of 56%. ^1^H NMR (400 MHz, DMSO, 25 °C, TMS) δ 11.91 (s, 1 H), 9.41 (t, J = 5.5 Hz, 1 H), 8.15 (d, J = 1.7 Hz, 1 H), 7.89-7.80 (m, 1 H), 7.79-7.72 (m, 1 H), 7.42 (dd, J = 8.4, 1.2 Hz, 1 H), 4.10 (dd, J = 5.5, 2.5 Hz, 1 H), 3.35 (s, 5 H); ^13^C NMR (100 MHz, DMSO, 25°C, TMS) δ 191.85, 181.46, 164.18, 161.83, 161.57, 141.26, 138.04, 134.05, 133.67, 125.01, 122.97, 119.92, 118.18, 118.01, 116.47, 81.20, 73.73, 40.52, 40.31, 40.11, 39.90, 39.69, 39.48, 39.27, 29.27. ESI-MS (positive mode, m/z): calcd. 322.07, found 322.33 for **rhein-alkyne**.

**Synthesis of Ru-N_3_**

4-Azidomethyl-4’-methyl-2, 2’-bipyridine was synthesized with the procedures reported as the references [1]. The dichloro(*p*-cymene) ruthenium(II) dimer (61.2 mg, 0.1 mmol) and 4-azidomethyl-4’-methyl-2, 2’-bipyridine (45 mg, 0.2 mmol) were dissolved in CH_3_OH (10 mL) under nitrogen. The mixture was stirred at room temperature overnight in dark. After the reaction completed, the solvent was removed under vacuum. Then the residue was dissolved in 5 mL CH_3_OH, 5-fold excess ammonium hexafluorophosphate was slowly added to the CH_3_OH solution, yielding yellow participate. The precipitate was collected and purified by column chromatography on silica gel (dichloromethane/methanol = 30/1) to give a yellow solid, with a yield of 43%. ^1^H NMR (400 MHz, CD_3_OD, 25 °C, TMS) δ 9.43 (d, J = 5.9 Hz, 1 H), 9.29 (d, J = 5.8 Hz, 1 H), 8.45 (d, J = 19.1 Hz, 2H), 7.68 (ddd, J = 44.9, 5.9, 1.4 Hz, 2 H), 6.15-6.05 (m, 2 H), 5.88-5.76 (m, 2 H), 4.83 (s, 2 H), 2.65 (m, 4 H), 2.28 (s, 3 H), 1.06 (dd, J = 6.9, 1.0 Hz, 6 H). ESI-MS (positive mode, m/z): calcd. 496.08, found 496.25 for [**Ru-N_3_** - PF_6_]^+^.

**Synthesis of Ru-rhein**

A mixture of **rhein-alkyne** (0.1 mmol), **Ru-N_3_** (0.1 mmol), CuI (0.02 mmol) and sodium ascorbate (0.02 mmol) in 5 mL DMF were stirred at room temperature under nitrogen in dark for 24 h. Then the reaction solvent was removed on a rotary evaporator. The precipitate was collected and purified by column chromatography on silica gel (dichloromethane / methanol = 20/1) to give a yellow solid, with a yield of 41%. ^1^H NMR (400 MHz, DMSO) δ 9.54 (s, 1H), 9.46 (dd, J = 24.7, 5.9 Hz, 1H), 9.34 (dd, J = 23.9, 5.8 Hz, 1H), 8.55 (s, 1H), 8.46 (d, J = 6.8 Hz, 1H), 8.30 (s, 1H), 8.18 (s, 1H), 7.84 (dd, J = 14.0, 5.9 Hz, 2H), 7.76 (d, J = 7.3 Hz, 1H), 7.64 (dd, J = 13.6, 5.8 Hz, 1H), 7.45 (dd, J = 22.5, 6.4 Hz, 2H), 6.19 (dd, J = 12.8, 6.1 Hz, 2H), 6.12 (t, J = 5.7 Hz, 1H), 5.96 (dd, J = 16.7, 8.4 Hz, 2H), 5.88 (s, 2H), 4.60 (d, J = 5.3 Hz, 2H), 2.58 (d, J = 3.9 Hz, 4H), 2.15 (s, 2H), 2.09 (s, 4H), 0.94 (t, J = 6.5 Hz, 5H). ESI-MS (positive mode, m/z): calcd. 817.15, found 817.33 for [**Ru-rhein** - PF_6_]^+^.

**Synthesis of Os-N_3_**

Synthesis of **Os-N_3_** was similar to that of **Ru-N_3_**. The dichloro(*p*-cymene) osmium(II) dimer (70.1 mg, 0.1 mmol) and 4-azidomethyl-4’-methyl-2,2’-bipyridine (45 mg, 0.2 mmol) were dissolved in CH_3_OH (10 mL) and reacted overnight at room temperature. Then the solvent was condensed to 5 mL and added with 5-fold excess ammonium hexafluorophosphate and purified by column chromatography on silica gel (dichloromethane/methanol = 15/1) with a yield of 50%. ^1^H NMR (400 MHz, MeOD) δ 9.38 (d, J = 6.0 Hz, 1H), 9.24 (d, J = 5.9 Hz, 1H), 8.58 (d, J = 1.1 Hz, 1H), 8.53 (s, 1H), 7.71 (dd, J = 6.0, 1.8 Hz, 1H), 7.60 (dd, J = 5.9, 1.1 Hz, 1H), 6.35-6.30 (m, 2H), 6.03-5.98 (m, 2H), 4.90 (s, 2H), 2.72 (s, 3H), 2.50 (dt, J = 18.0, 6.9 Hz, 1H), 2.35 (s, 3H), 1.00 (d, J = 6.9 Hz, 6H).

**Synthesis of Os-rhein**

Synthesis of **Os-rhein** was similar to that of **Ru-rhein**. A mixture of **rhein-alkyne** (0.1 mmol), **Ru-N_3_** (0.1 mmol), CuI (0.02 mmol) and sodium ascorbate (0.02 mmol) in 5 mL DMF were stirred at room temperature under nitrogen in dark for 8 h. Then the reaction solvent was removed on a rotary evaporator. The precipitate was collected and purified by column chromatography on silica gel (dichloromethane/methanol = 8/1) to give a red solid, with a yield of 38%. ^1^H NMR (400 MHz, MeOD) δ 9.36 (d, J = 5.9 Hz, 1H), 9.22 (d, J = 5.9 Hz, 1H), 8.48 (d, J = 19.1 Hz, 2H), 8.24 (s, 1H), 8.16 (s, 1H), 7.86-7.68 (m, 4H), 7.58 (d, J = 5.8 Hz, 1H), 7.49 (d, J = 5.6 Hz, 1H), 7.39 -7.32 (m, 1H), 6.29 (dd, J = 9.5, 5.9 Hz, 2H), 6.06-5.91 (m, 4H), 4.72 (s, 2H), 2.69 (s, 3H), 2.47 (dt, J = 13.6, 6.8 Hz, 1H), 2.34-2.28 (m, 3H), 0.96 (t, J = 7.7 Hz, 6H). ESI-MS (positive mode, m/z): calcd. 907.21, found 907.50 for [**Os-rhein** - PF_6_]^+^.

**Synthesis of Ir(Cp*)-N_3_**

The procedure of **Ir(Cp*)-N_3_** was similar to that of **Ru-N_3_**. The pentamethylcyclopentadienyl)iridium(III) chloride dimer (79.6 mg, 0.1 mmol) and 4-azidomethyl-4’-methyl-2,2’-bipyridine (45 mg, 0.2 mmol) were reacted in CH_3_OH overnight at room temperature and then added with 5-fold excess ammonium hexafluorophosphate, then purified by column chromatography on silica gel (dichloromethane/ methanol = 15/1) with a yield of 57%. ^1^H NMR (400 MHz, DMSO) δ 8.95 (d, J = 5.9 Hz, 1H), 8.82 (d, J = 5.8 Hz, 1H), 8.72 (d, J = 3.0 Hz, 2H), 7.80 (d, J = 5.8 Hz, 1H), 7.71 (d, J = 5.7 Hz, 1H), 5.76 (s, 1H), 4.95 (s, 2H), 2.64 (s, 3H), 1.66 (d, J = 4.7 Hz, 15H).

**Synthesis of Ir(Cp*)-rhein**

Synthesis of **Ir(Cp*)-rhein** was similar to that of **Ru-rhein**. A mixture of **rhein-alkyne** (0.1 mmol), **Ir(Cp*)-N_3_** (0.1 mmol), CuI (0.02 mmol) and sodium ascorbate (0.02 mmol) in 5 mL DMF were stirred at room temperature under nitrogen in dark for 12 h. Then the reaction solvent was removed on a rotary evaporator. The precipitate was collected and purified by column chromatography on silica gel (dichloromethane/ methanol = 10/1) to give a red solid, with a yield of 47%. ^1^H NMR (400 MHz, DMSO) δ 11.92 (d, J = 12.3 Hz, 2H), 9.56 (t, J = 5.3 Hz, 1H), 8.95 (d, J = 5.9 Hz, 1H), 8.82 (d, J = 5.8 Hz, 1H), 8.69 (s, 1H), 8.59 (s, 1H), 8.32 (s, 1H), 8.19 (s, 1H), 7.85 (dd, J = 14.0, 5.9 Hz, 2H), 7.77 (d, J = 7.2 Hz, 1H), 7.67 (d, J = 5.3 Hz, 1H), 7.44 (d, J = 7.6 Hz, 2H), 6.00-5.93 (m, 2H), 4.63 (t, J = 8.8 Hz, 2H), 2.64 (d, J = 7.0 Hz, 3H), 1.79-1.67 (m, 15H). ESI-MS (positive mode, m/z): calcd. 909.21, found 909.33 for [**Ir(Cp*)-rhein** - PF_6_]^+^.

**UV-vis and Photoluminescence spectroscopic study**

The stock solutions (2 mM) of **rhein-alkyne** and **Ru-rhein** were prepared with DMSO and stored in a 4 ^o^C refrigerator. The absorption and fluorescence spectra of **rhein-alkyne** (10 μM) and **Ru-rhein** (10 μM) were determined in PBS buffer (20 mM, pH 7.40) containing 5% DMSO at ambient temperature.

**Measurement of partition coefficients (log *P*_o/w_)**

The lipophilicity/hydrophilicity of **Ru-N_3_** and **Ru-rhein** was assessed by calculated the octanol/water partition coefficient (log*P*_o/w_). The detailed procedures were as follows: Pre-saturated PBS buffer and octanol were obtained by shaking the mixture of PBS buffer and octanol for 7 days. Then the pre-saturated octanol (2 mL) containing **Ru-N_3_** or **Ru-rhein** was reacted with PBS (2 mL) in 10 mL tube, respectively and shaken in dark for 4 h at room temperature. Then two phases were separated by centrifugation and the concentrations of **Ru-N_3_** or **Ru-rhein** in the two phases were determined by spectrophotometry. The related partition coefficients were calculated by the equation log *P*_o/w_ = log (A_o_/A_w_), where "A" referred to the absorbance of the complex at maximum absorption. The final results were expressed based on the data of three independent tests.

**Cellular confocal images**

A549 cells were plated on 35 mm dishes in DMEM medium (containing 10% fetal bovine serum, FBS) in an atmosphere of 5% CO_2_ and 95% air at 37 °C. Then the cells were separately incubated with **rhein-alkyne** and chemical synthesized **Ru-rhein** (10 μM) for 12 h, then the solution was suck out, washed with PBS for three times and co-stained with Mito-Tracker Deep Red at 37 °C for another 0.5 h. The cells were washed twice with PBS buffer solution and visualized immediately using confocal microscopy with a 63×oil-immersion objective lens for confocal imaging. For **rhein-alkyne** and **Ru-rhein**-incubated cells, a band path of 560-650 nm upon excitation at 488 nm was adopted; the excitation wavelength of Mito Tracker-Deep Red is 633 nm with band path of 660-740 nm.

For the imagings of **Ru-rhein**-induced autophagy stained by MDC, A549 cells were plated on 35 mm dishes in DMEM medium (containing 10% fetal bovine serum, FBS) in an atmosphere of 5% CO_2_ and 95% air at 37 °C. The cells were separately incubated with 10 μM **Ru-rhein** and chemical synthesized **Ru-rhein** for 24 h, and then washed with PBS for three times and co-stained with 0.05 mM MDC in PBS at 37 °C for 20 min. After that, the cells were washed four times with PBS and immediately analyzed using confocal microscopy with a 63×oil-immersion objective lens for imaging. The excitation of MDC was 405 nm with the emission bandpath of 430-460 nm.

***In vitro* cytotoxicity assays**

The cytotoxicities of the complexes toward four cancer cell lines A549, A2780, MCF-7, two normal HLF and LO2 cells lines were determined *via* MTT assays. The cell lines were separately cultured in 96-well plates in DMEM medium (both containing 10% heat-inactivated fetal bovine serum (FBS) and 100 U mL^‒1^ penicillin) in 5% CO_2_ atmosphere at 37 °C. Solutions of the compounds **rhein-alkyne**, **Ru-N_3_**, **Ru-rhein** (mixture of **rhein-alkyne**/**Ru-N_3_** with different mole ratios: 1/1, 2/1 and 1/2) and cisplatin (0-100 μM) were separately added into the wells and incubated for 48 h at 37 °C. Then an amount of 20 μL MTT [3-(4, 5-dimethylthiazol-2-yl)-2, 5-diphenyltetrazolium bromide] (5 mg mL^‒1^, PBS buffer) was added to each well and incubated for another 4 h. The medium was carefully removed and dissolved in DMSO (150 μL per well) was added and the absorbance at 570 nm of the purple formazan was collected on a Varioskan Flash microplate reader (Thermo Scientific). The cell viabilities were calculated based on the data of three parallel tests.

Moreover, to clarify the effect of the ROS species to the cytotoxicity of **Ru-rhein**, A549 cells were cultured in 96-well plates and pre-incubated with 5 mM ROS scavenger N-Acetyl-L-cysteine (NAC) for 2 h. Then different concentrations of **Ru-rhein** were added to the cells and incubated for another 46 h. The 48 h-cell viability was determined and calculated based on the data of three parallel tests.

**Cellular ESI-MS detection**

A549 cells were seeded in 6 cm dishes with a density of 2×10^5^ cells/mL for 24 h at 37 °C, and then incubated with **Ru-rhein** (a mixture of 10 μM **rhein-alkyne** and **Ru-N_3_**) or chemical synthesized **Ru-rhein** (10 μM) for 12 h, respectively. Then the cells were harvested with 0.25% trypsin (Gibco), washed with PBS three times. After that, the cells were broken with an ultrasonic cell disruptor every two seconds for 20 min on the ice with 20% power. Then the ESI-MS data were collected with an LCQ electrospray mass spectrometer (Thermo Finnigan) and analyzed on the Xcalibar software.

**Cellular uptake of complexes**

A549 cells were seeded in 6-well plates with a density of 10^5^ cells/mL for 24 h at 37 °C in a humidified atmosphere of 5% CO_2_, and then incubated with the complexes **Ru-N_3_** (10 μM) and **Ru-rhein** (10 μM) for different time (4, 8 and 12 h, respectively). Then the cells were harvested with 0.25% trypsin (Gibco), washed with PBS three times. The Mitochondria /Nuclei Isolation Kit (Beyotime Biotechnology) was added to the cell samples, and kept in an ice bath for 15 min. After that, the cell suspension was homogenized for about 30 times and centrifuged at 800 g for 5 min at 4 ^o^C, the nuclei are distributed in the precipitate, while the mitochondria are distributed in the supernatant. Then Medium Buffer A, B and C were added to obtain purified nuclei, mitochondria and cytosol samples. The above nuclei, mitochondria and cytosol samples were separately digested successively with concentrated nitric acid (65%, 100 μL) at 95 ^o^C for 2 h, hydrogen peroxide (30%, 50 μL) at 95 ^o^C for 1.5 h, and concentrated hydrochloric acid (37%, 20 μL) at 37 ^o^C for 0.5 h. The resulting samples were diluted to 1 mL with water from Milli-Q system (>18 MΩ) and tested on the inductively coupled plasma mass spectrometer (ICP-MS, VG Elemental). The result shown in Figure is the mean of three experiments and presented as the mean ± SD.

**Cellular uptake of endogenous copper species**

A series of cells such as A2780, A549, MCF-7 (cancer cells) and normal LO2, HLF cells were separately seeded in 6-well plates with a density of 10^6^ cells/mL and cultured for 24 h. Then the cells were separately harvested with 0.25% trypsin (Gibco), rinsed twice with PBS, and digested successively with concentrated nitric acid (65%, 100 μL) at 95 ^o^C for 2 h, hydrogen peroxide (30%, 50 μL) at 95 ^o^C for 1.5 h, and concentrated hydrochloric acid (37%, 20 μL) at 37 ^o^C for 0.5 h. The resulting samples were diluted to 1 mL with water from Milli-Q system (>18 MΩ) for copper determination using an inductively coupled plasma mass spectrometer (ICP-MS, VG Elemental). The experiment was carried out in triplicate.

**Study on intracellular generation of ROS**

A549 cells were plated on 35 mm dishes for 24 h and treated with **Ru-rhein** and chemical synthesized **Ru-rhein** (0, 5 and 10 μM) separately for 12 h, then they were washed with 1×PBS twice and incubated with 5 μM fluorescent probe DCFH-DA for another 20 min at 37 °C. After that, DCFH-DA was removed from the cells and analyzed immediately by confocal microscopy (Nikon TI-E-A1R, Japan) using a 60× oil lens with the emission at 525 nm (λ_ex_=488 nm). The images were captured by confocal fluorescence microscopy and processed using the NIS-Elements Viewer.

For the flow cytometry analysis of cellular ROS, A549 cells were seeded in 6-well plates for 24 h, incubated with **Ru-rhein** with different concentrations of 0, 5 and 10 μM for 12 h. Then the cells were collected and washed with PBS twice. After that, the ROS probe DCFH-DA were added to the cell samples and incubated for 20 min. Then the cell samples were analyzed by flow cytometry after washing with culture without FBS and analyzed using a BD FACS Calibur flow cytometer.

**Mitochondrial membrane potential detection**

A549 cells were seeded in 6-well plates for 24 h, incubated with **Ru-rhein** with different concentrations of 0, 5 and 10 μM for 12 h. Then the cells were harvested with 0.25% trypsin (Gibco), washed with PBS three times. After the addition of 0.5 mL JC-1 working solution, the cells were incubated at 37 °C for 20 min. Then the staining solution was removed, the cell samples were washed with 1× incubation buffer and analyzed by flow cytometry using a BD FACS Calibur flow cytometer.

**Expression of autophagy-related protein by Western Blot Analysis**

A549 Cells were cultured in 10 cm dishes and separately treated with 10 μM **Ru-rhein** and chemical synthesized **Ru-rhein** for 24 h, and harvested in a 1.5 mL microcentrifuge tube for each sample. Then the proteins were extracted in lysis buffer (KeyGen Biotech), run on SDS-PAGE gels (Amresco) and transferred to PVDF membranes (Millipore). The membranes were incubated with specific antibodies at 4 °C overnight, and then incubated with a horse radish peroxidase-coupled secondary antibody for 1 h at room temperature. After washing with PBST, the immunoblots were visualized by chemiluminescence using a HRP substrate (Millipore).

**Transmission electron microscopy assay**

1×10^6^ A549 Cells were cultured in 10 cm dishes and separately treated with 10 μM **Ru-rhein** and chemical synthesized **Ru-rhein** for 24 h, and harvested in a 1.5 mL microcentrifuge tube for each sample. Then the cells were washed with cold PBS twice and fixed with cold fixation solution (2.5% glutaraldehyde in 0.1 mol/L phosphate buffer) at 4 °C overnight. After that, the cell samples were dehydrated, fixed, embedded and sliced, finally observed using transmission electron microscopy (Hitachi).

**Cell cycle analysis**

A549 cell lines were maintained in Dulbecco’s modified Eagle’s medium with 10% fetal bovine serum in 5% CO_2_ at 37 ^o^C and then treated with **Ru-rhein** (0-20 μM). Then the cells were harvested by trypsin and rinsed with PBS. After centrifugation, the pellet (10^5^-10^6^ cells) was suspended in 1 mL of PBS and kept on ice for 5 min. The cell suspension was then fixed by the dropwise addition of 9 mL precooled (4 ^o^C) 70% ethanol with violent shaking. Fixed samples were kept at 4 ^o^C overnight. For staining, cells were centrifuged, resuspended in PBS, digested with 150 mL of RNase A (250 mg/mL), and treated with 5 μL of propidium iodide (PI), then incubated for 30 min at 4 ^o^C. Then the cell samples were detected on flow cytometry and analyzed a BD FACS Calibur flow cytometer.

**Induction of apoptosis**

The ability of **Ru-rhein** to induce apoptosis was evaluated in the A549 cell line using flow cytometry. Cells in the exponential growth phase were cultured for 24 h in 6-well plates and then treated with **Ru-rhein** (0, 5 and 10 μM). Then the cells were harvested after 24 h-incubation, washed with PBS twice, and re-suspended in 100 μL binding buffer at a final concentration of 1×10^6^ cells / mL. The resuspended cells were further incubated with 5 μL annexin V-FITC and 10 μL propium iodide (PI) (20 μg / mL) for 20 min at room temperature in the dark. The cells were analyzed by flow cytometry using a BD FACS Calibur flow cytometer.

***In vivo* anticancer activity**

The *in vivo* anticancer activity studies were carried out in A549 tumor-bearing nude mice models. All mice were purchased from Yangzhou University (5-week-old, female, n=30). A549 cells were inoculated subcutaneously in the back of mice. The mice were randomized into 5 groups (6 mice/group) when the tumors grew to 100-150 mm^3^. The groups were injected with various compounds of **Ru-rhein**, **rhein-alkyne**, **Ru-N_3_**, saline and chemical synthesized **Ru-rhein** (8.0 mg kg^‒1^ body weight, dissolved in containing 0.9% saline solution) once every three days for 18 days (0, 3, 6, 9, 12 and 15), respectively. The volume of tumor was monitored by Vernier caliper and calculated according to the following formula: Volume=width^2^×length×0.5. The body weight of mice was measured at the same time.

After the mice executed at 18^th^ day after treatment, the tumors and the organisms such as heart, liver, lung, spleen and kidney were collected and fixed in formalin for paraffin embedding. Hematoxylin and eosin (H&E), and the TUNEL apoptosis staining of the formalin-fixed tumors were prepared by Wuhan Servicebio Technology Co. Ltd. and observed by fluorescence microscopy (NIKON ECLIPSE Ci-L).

**Statistical analysis**

Data are given as Mean ± SD. Statistical significance was performed using two-tailed Student’s *t*-test. Statistical significance was set at ^*^*P* < 0.05, and extreme significance was set at ^**^*P* < 0.01, and ^***^*P* < 0.001.

**Scheme S1.** Design and synthesis of the compounds.


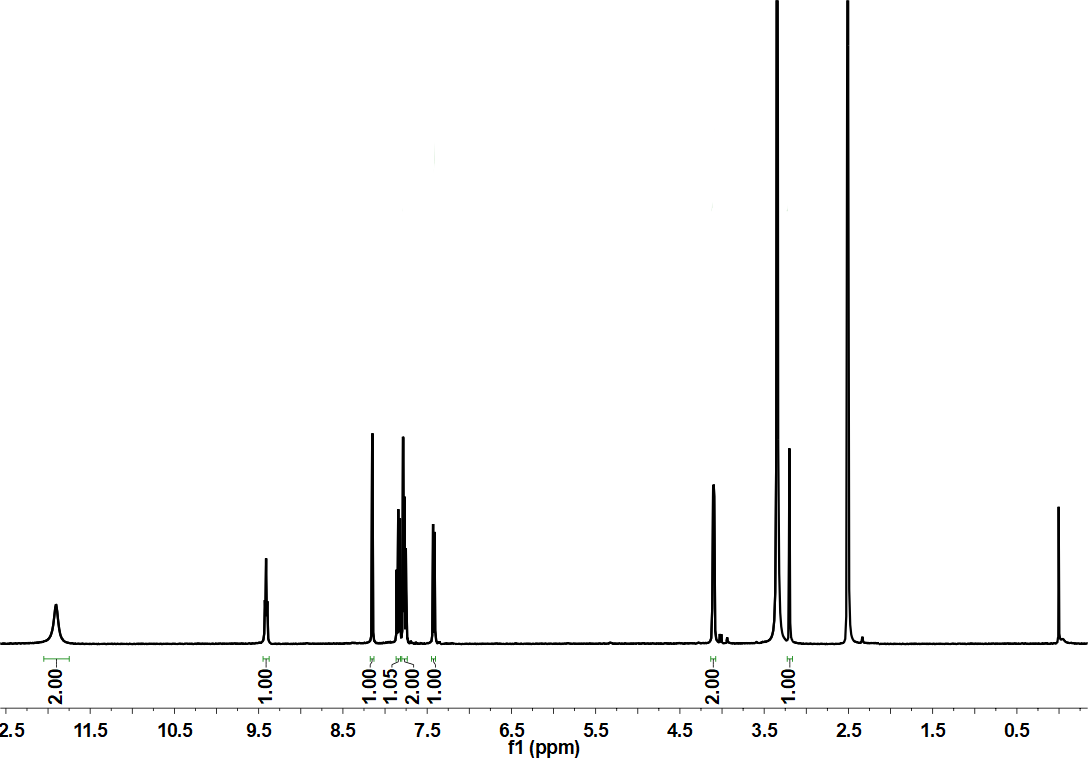


**Figure S1.** ^1^H NMR Data of **rhein-alkyne** in d_6_-DMSO.

**
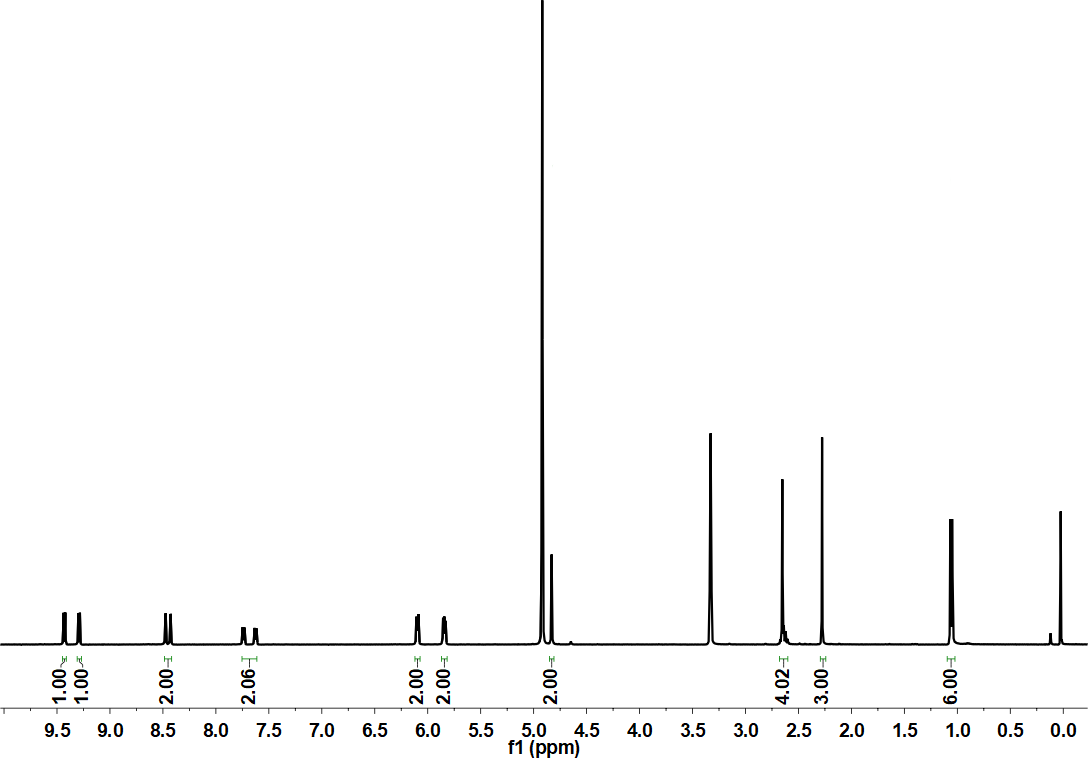
**

**Figure S2.** ^1^H NMR Data of **Ru-N_3_** in CD_3_OD.


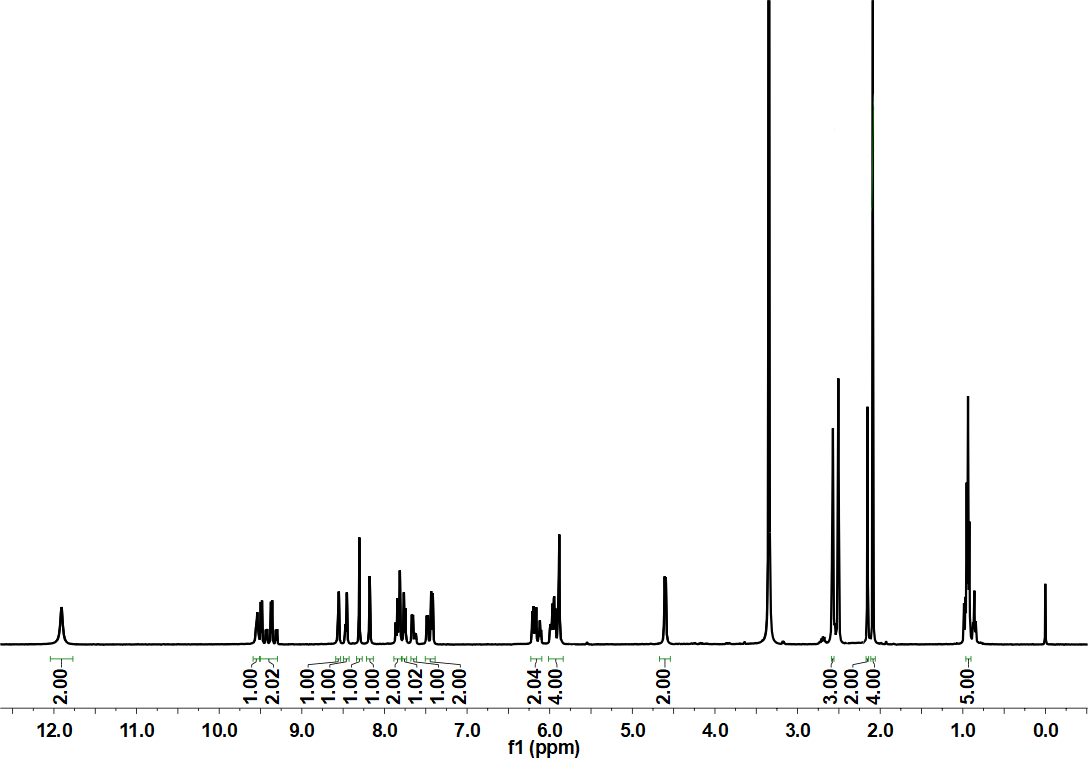


**Figure S3.** ^1^H NMR Data of **Ru-rhein** in d_6_-DMSO.

**
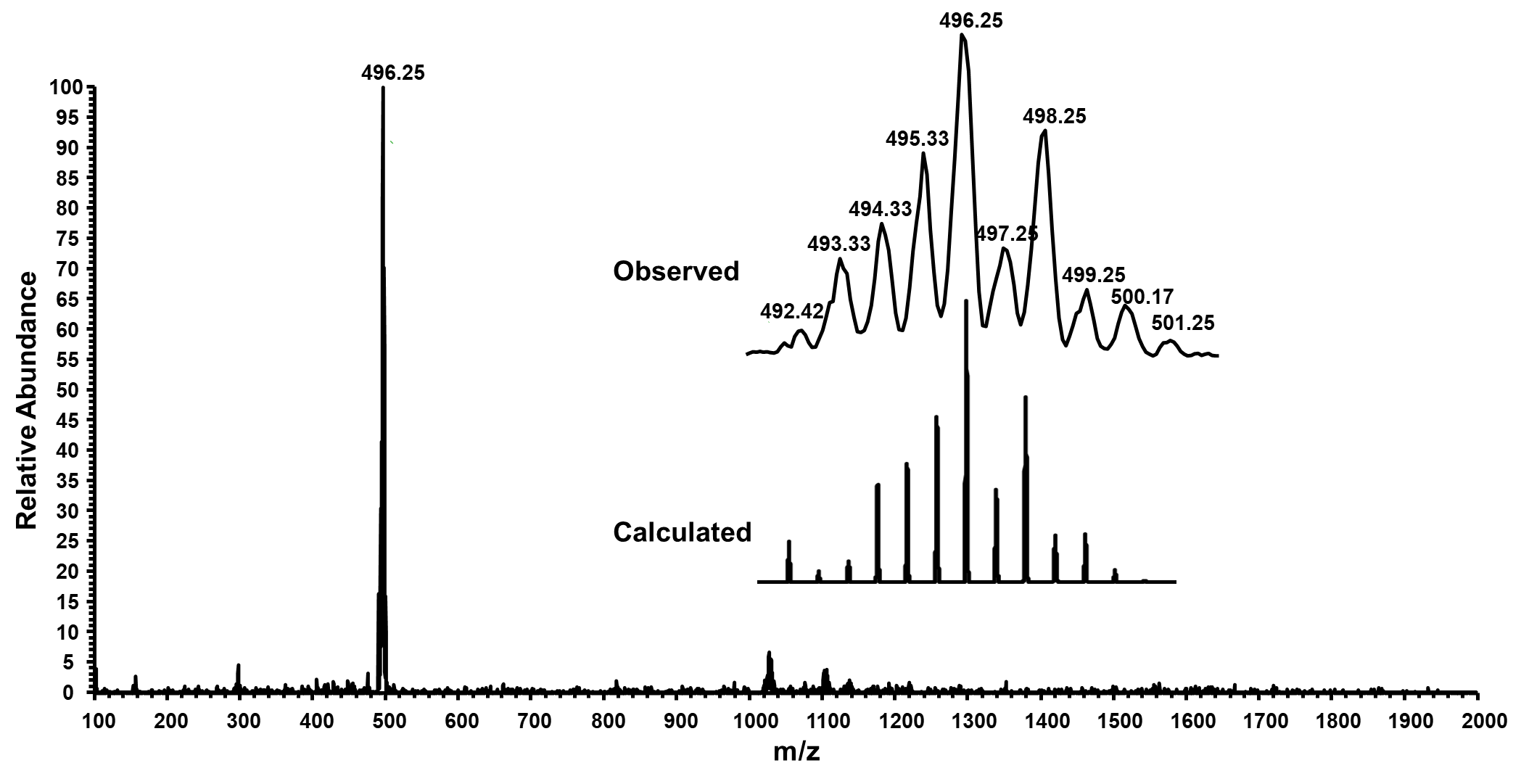
**

**Figure S4.** The ESI-MS data of **Ru-N_3_** in CH_3_OH solution. The observation of the m/Z peak 496.25 was consistent with the calculated results [**Ru-N_3_ -** PF_6_]^+^ (496.08).


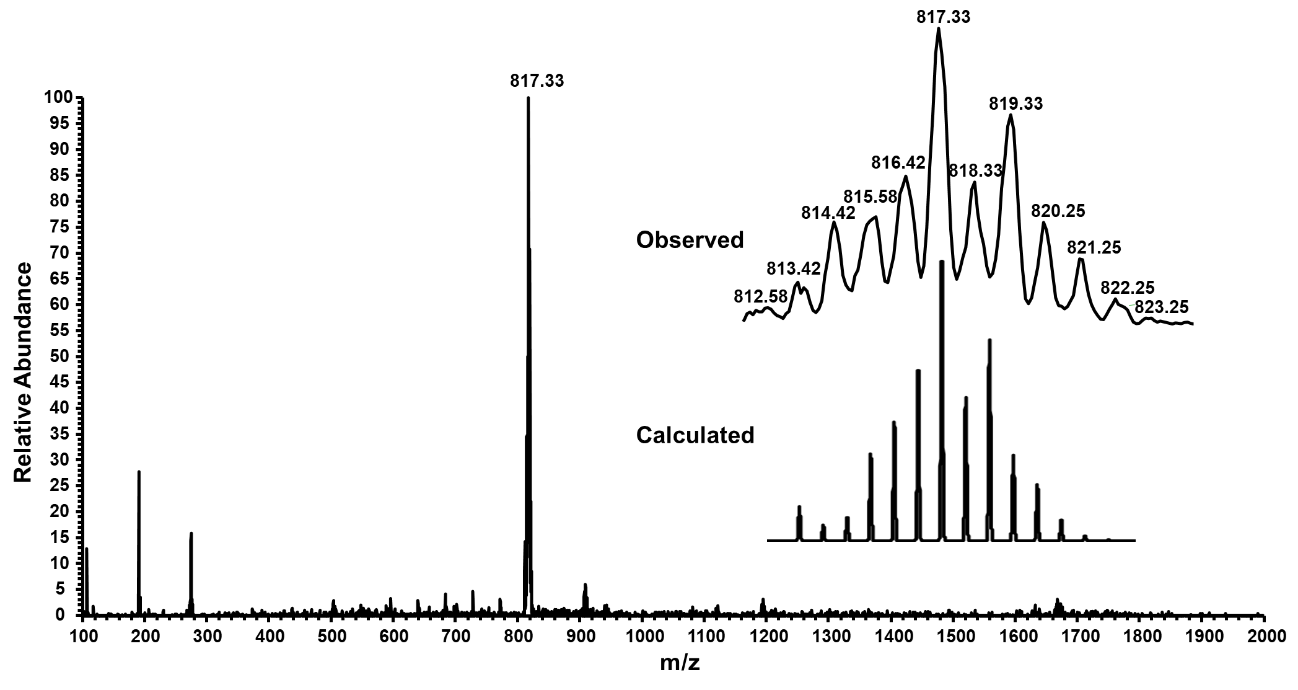


**Figure S5.** The ESI-MS data of chemical synthesized **Ru-rhein** in CH_3_OH solution. The observation of the m/Z peak 817.33 was consistent with the calculated results [**Ru-rhein** - PF_6_]^+^ (817.15)


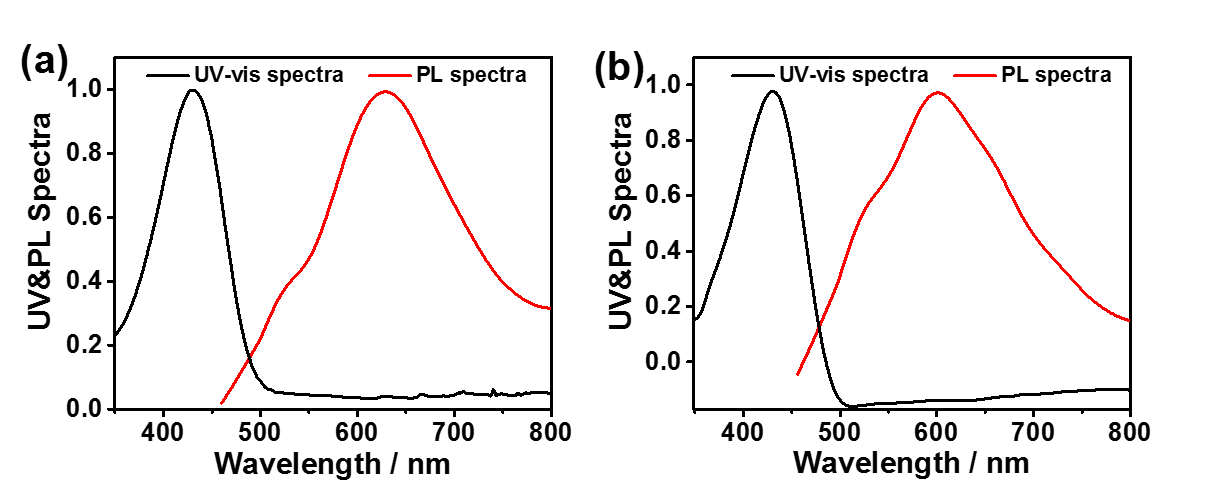


**Figure S6.** UV-vis spectra and photoluminescence spectra for **rhein-alkyne** (a) and **Ru-rhein** (b) in PBS solution (20 mM, containing 5% DMSO).


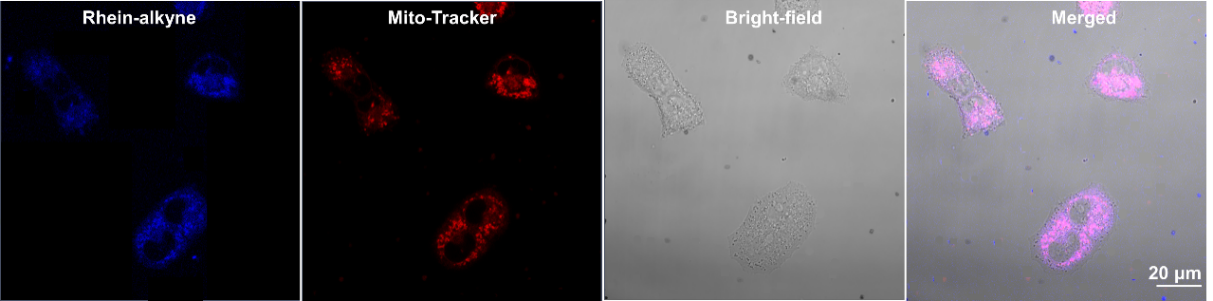


**Figure S7.** Confocal microscopy images of A549 cells incubated with **rhein-alkyne** (10 μM) for 4 h, then co-localized with Mito-Tracker Deep Red. The Pearson correlation coefficients were 0.89, respectively. λ_ex_=405 nm, λ_em_=520-700 nm for **rhein-alkyne**; λ_ex_=543 nm, λ_em_= 580-700 nm for Mito-Tracker. Scale bar: 20 μm.

**
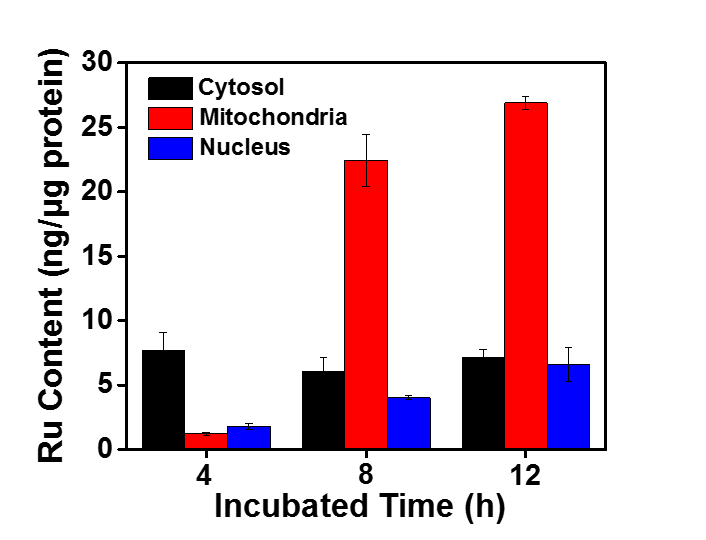
**

**Figure S8**. The ruthenium concentrations after treated with 10 μΜ **Ru-N_3_** within different organelles for different incubation time using ICP-MS technique.


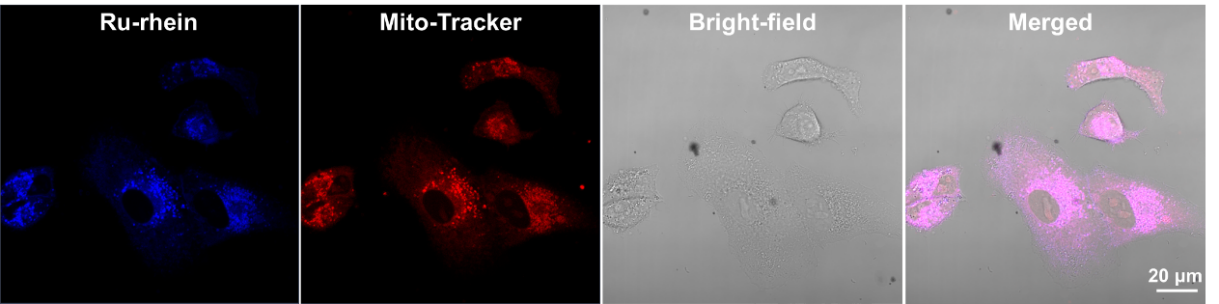


**Figure S9**. Confocal microscopy images of A549 cells incubated with chemical synthesized **Ru-rhein** (10 μM) for 12 h, then co-localized with Mito-Tracker Deep Red. The Pearson correlation coefficients was 0.86, indicating the mitochondrial-targeted ability of chemical synthesized **Ru-rhein**. λ_ex_=405 nm, λ_em_=520-700 nm for **Ru-rhein**; λ_ex_=543 nm, λ_em_= 580-700 nm for Mito-Tracker. Scale bar: 20 μm.


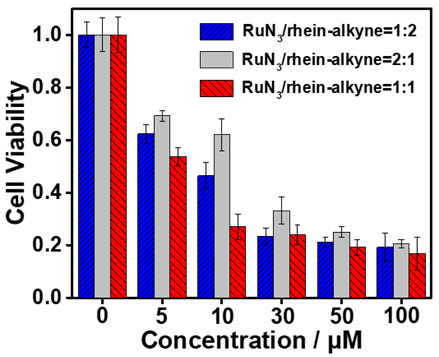


**Figure S10.** The cytotoxicities against A549 cells after treated with different ratio of **Ru-N_3_** and **rhein-alkyne** for 48 h. The cell viability proved that ratio of 1:1 between **Ru-N_3_** and **rhein-alkyne** was more effective in this experiment.


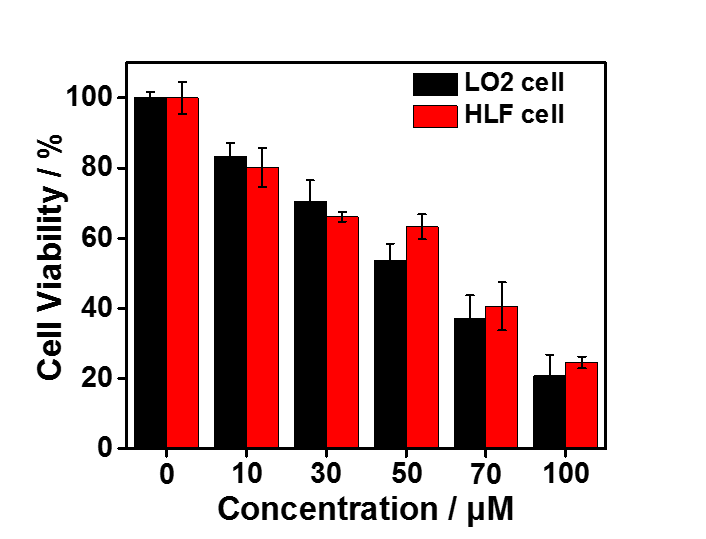


**Figure S11**. The cytotoxicities of **Ru-rhein** against the normal LO2 and HLF cells after 48 h of dark incubation.

**
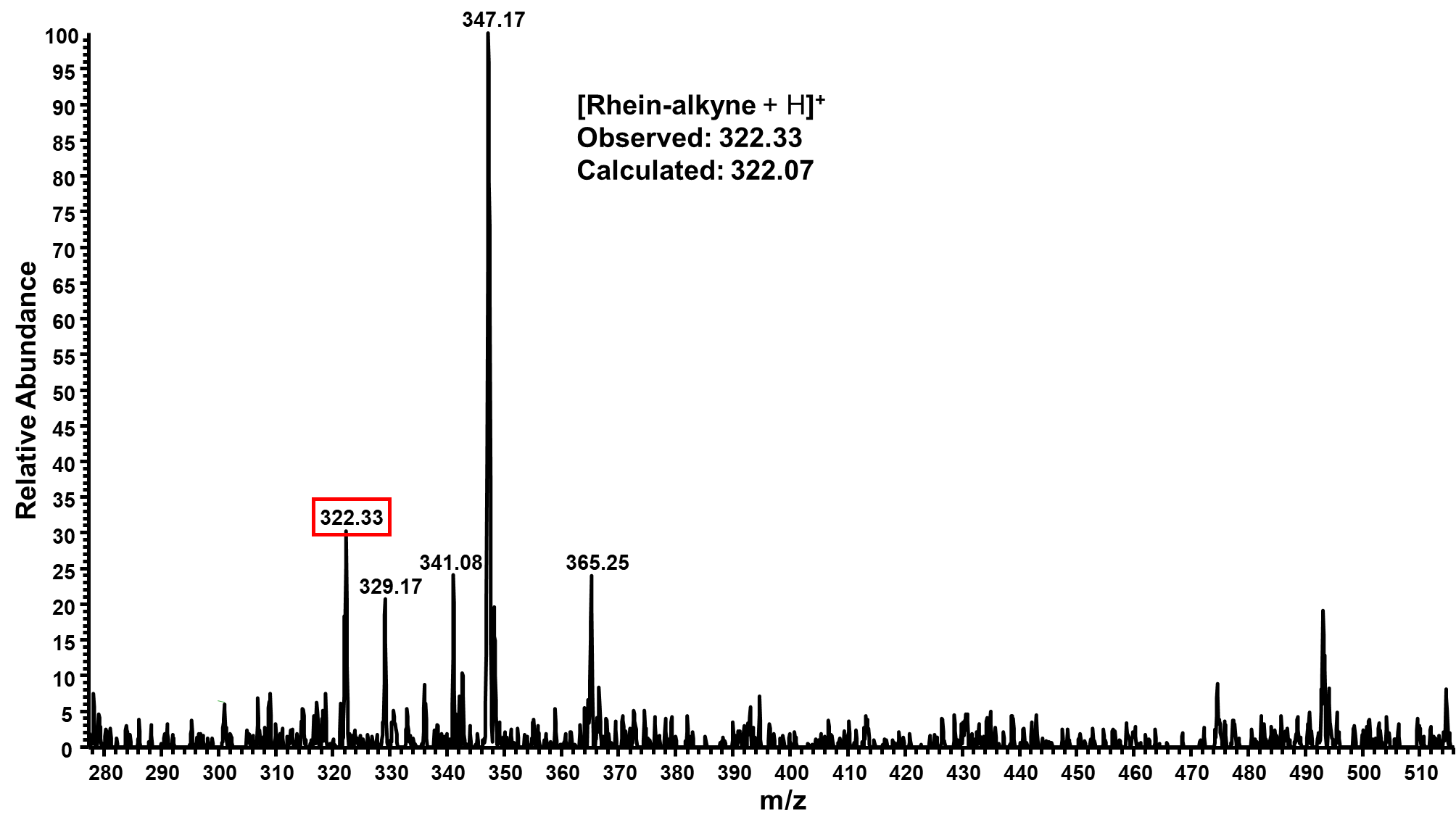
**

**Figure S12.** The ESI-MS results of A549 cellular sample incubated with **rhein-alkyne** (5 μM) for 12 h. The observation of the m/Z peak 322.33 was consistent with the calculated results (322.07).

**
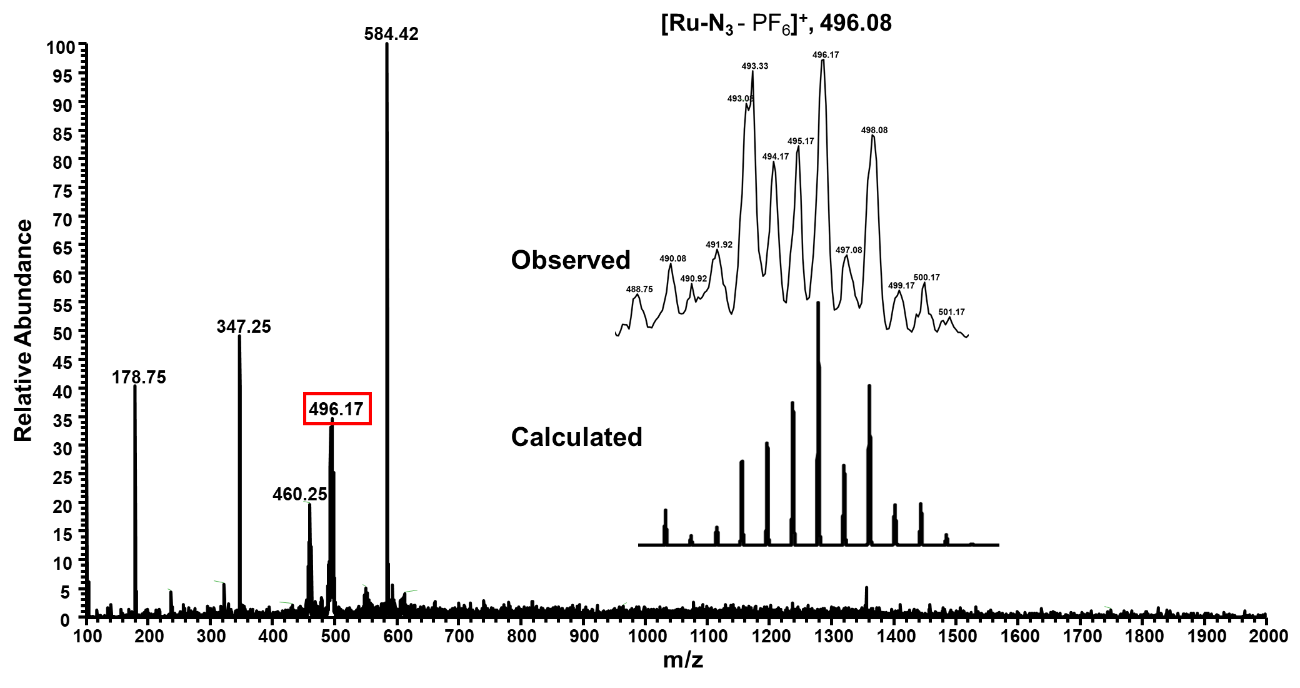
**

**Figure S13.** The ESI-MS results of A549 cellular sample incubated with **Ru-N_3_** (5 μM) for 12 h. The observation of the m/Z peak 496.17 was consistent with the calculated results (496.08).


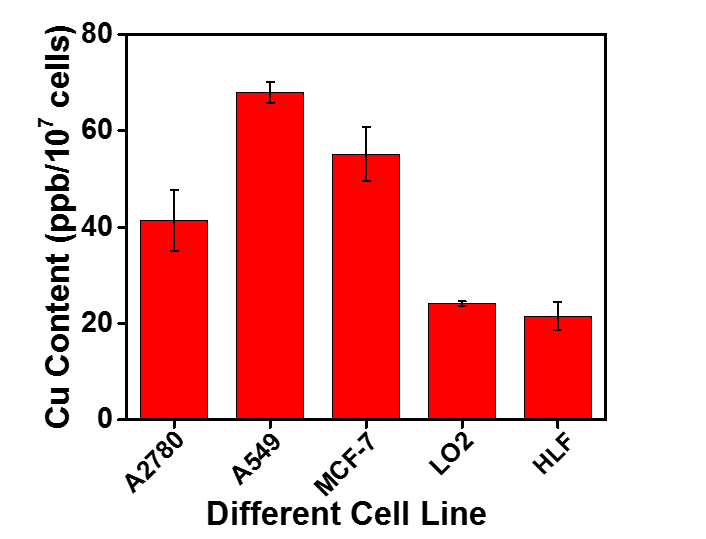


**Figure S14**. The copper concentrations in different cell lines using ICP-MS technique.

**Table S1**. The concentration of copper species in different cancer cells and the related IC_50_ values of **Ru-rhein** against them.

| **Cell Line** | A2780 | A549 | MCF-7 | LO2 | HLF |
| --- | --- | --- | --- | --- | --- |
| **Cu content (ppb/10^7^ cells)** | 41.4±6.4 | 67.9±2.2 | 55.1±5.6 | 24.2±0.5 | 21.6±2.9 |
| **IC_50_ value (μM)** | 10.5±0.7 | 5.6±1.6 | 12.1±0.9 | 53.9±4.8 | 67.3±1.4 |


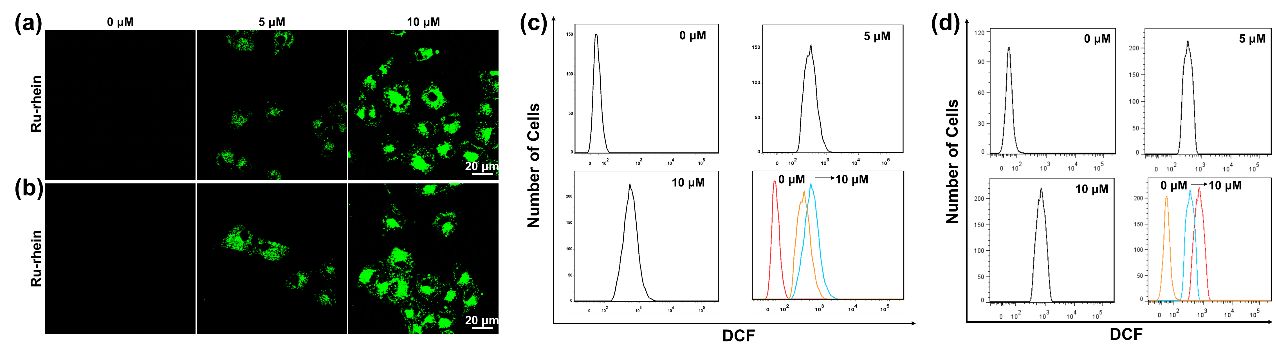


**Figure S15.** ROS generation in A549 cells treated with different concentrations of **Ru-rhein** (0-10 μM) stained with DCFH-DA as a probe, and then analyzed by fluorescence microscopy **(a)** and flow cytometry **(c)**, as the chemical synthesized **Ru-rhein** as the control group **(b** and **d)**. λ_ex_=488 nm; λ_em_=500 nm-540 nm for DCF. Scale bar: 20 μm.

**
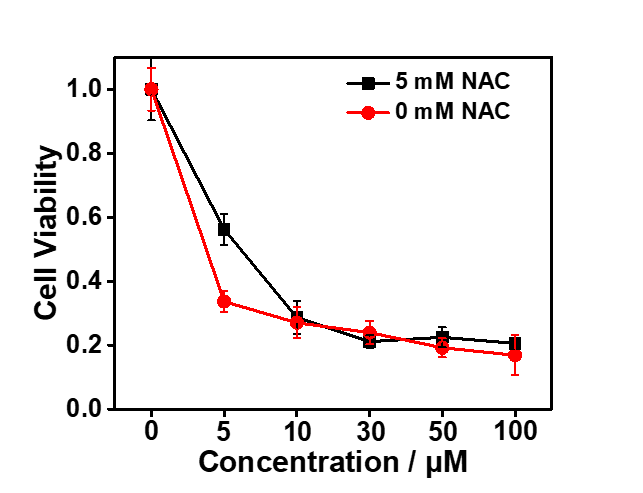
**

**Figure S16**. The 48 h-viability of A549 cells treated with **Ru-rhein** when pre-incubated with or without 5 mM NAC for 2 h. The similar IC_50_ data suggested that pre-treated with NAC (pre-treated with 5 mM NAC was 7.6±1.7 μM, without NAC was 5.6±1.6 μM) did not affect the cytotoxicity towards cancer cells, indicating the generation of ROS would hardly affect the activity of the endogenous copper catalysts and then the bioorthogonally catalytic lethality.


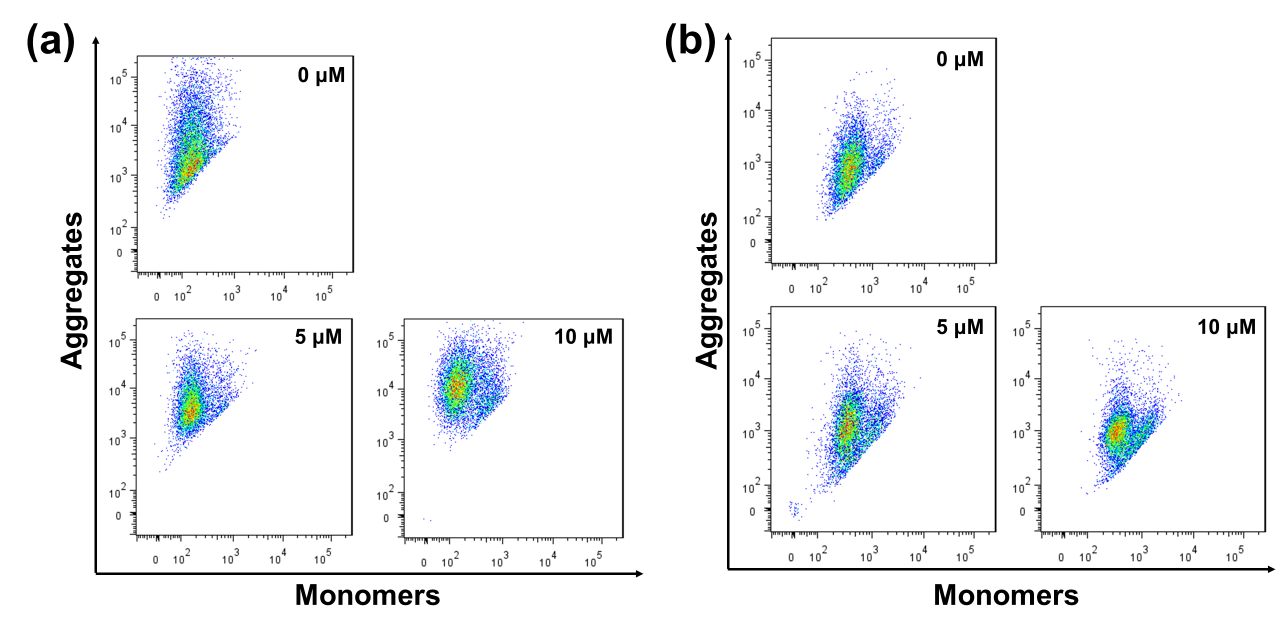


**Figure S17.** The loss of mitochondrial membrane potential in A549 cells stained with JC-1 was examined by flow cytometry after incubation with 0-10 μM **Ru-rhein (a)** for 24 h, as the chemical synthesized **Ru-rhein** as the control group **(b)**.


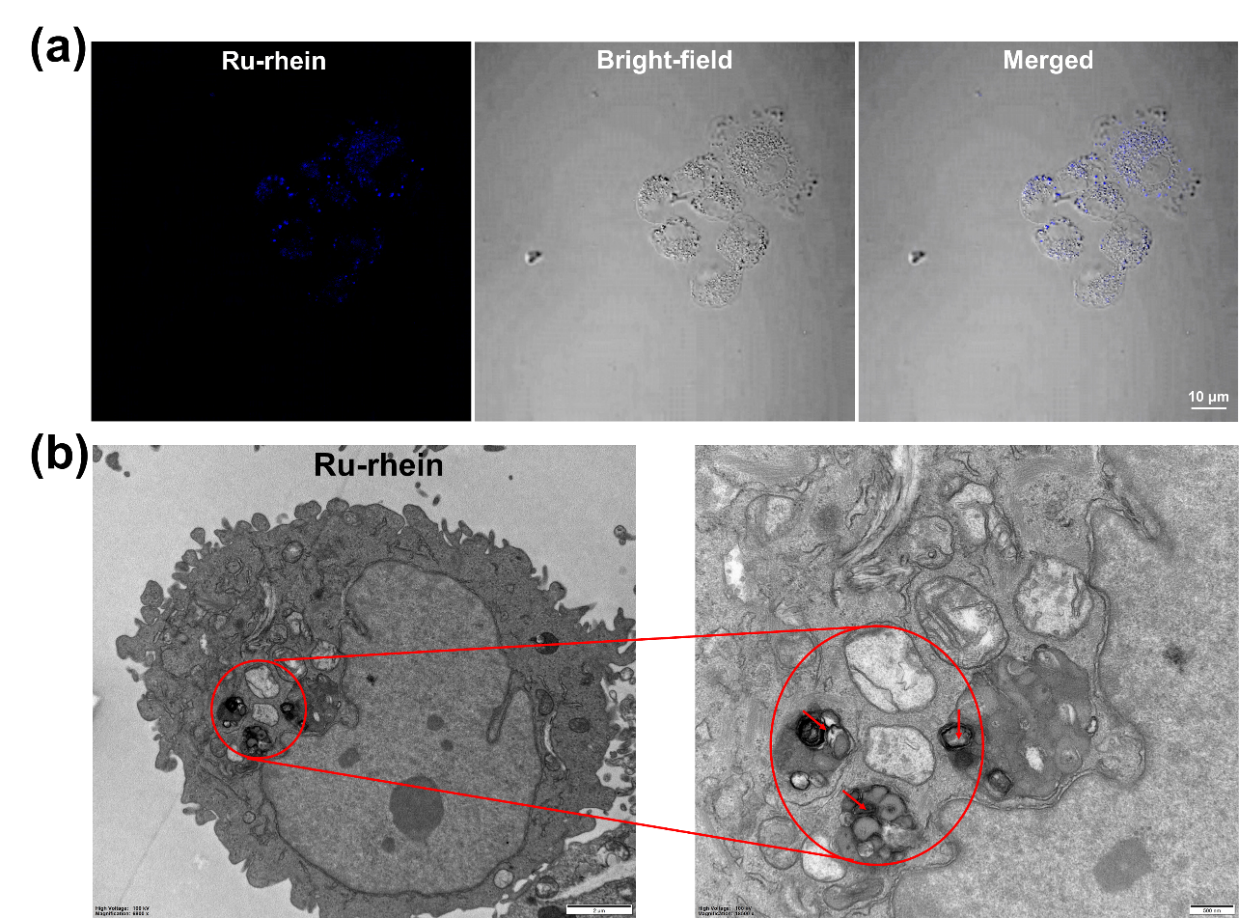


**Figure S18**. The cell death mechanism after treatment with the chemical synthesized **Ru-rhein**. **(a)** A549 cells were stained with 5 μM monodansylcadaverine (MDC) for 20 min after treatment with **Ru-rhein** (5 μM) separately for 24 h and then analyzed by fluorescence microscopy. The increased fluorescence indicated the **Ru-rhein**-induced formation of autophagosomes. λ_ex_=405 nm, λ_em_=430-600 nm for MDC. Scale bar: 10 μm. **(b)** Transmission electron microscopy imagings showed numerous autophagic vacuoles (red arrows) in A549 cells after treated with 5 μM **Ru-rhein** for 24 h.


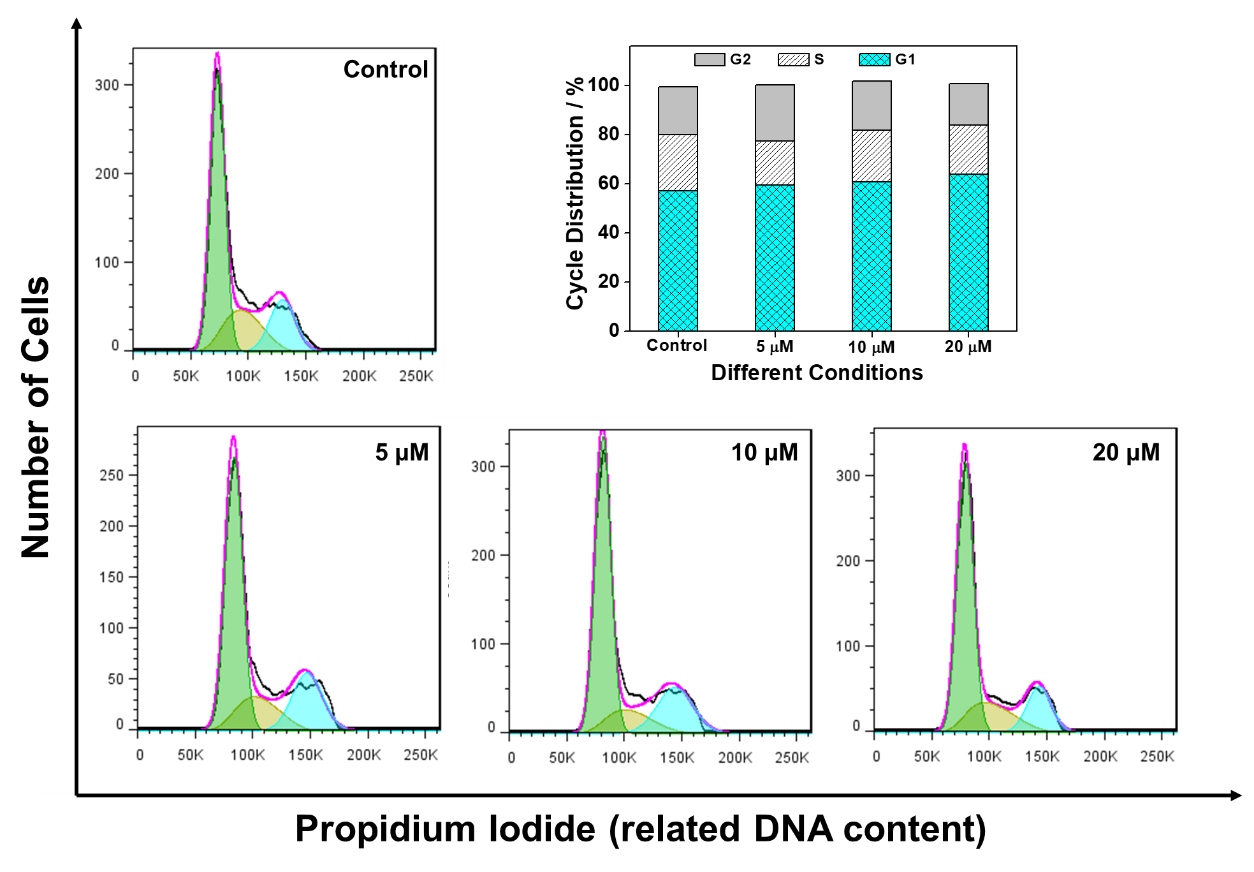


**Figure S19.** Effect of **Ru-rhein** on cell cycle progression in A549 cells. A549 cells were exposed to the indicated concentrations of **Ru-rhein** (0-20 μM) for 24 hours. The cell cycle distributions were analyzed by flow cytometry as described in the Materials and Methods.

**
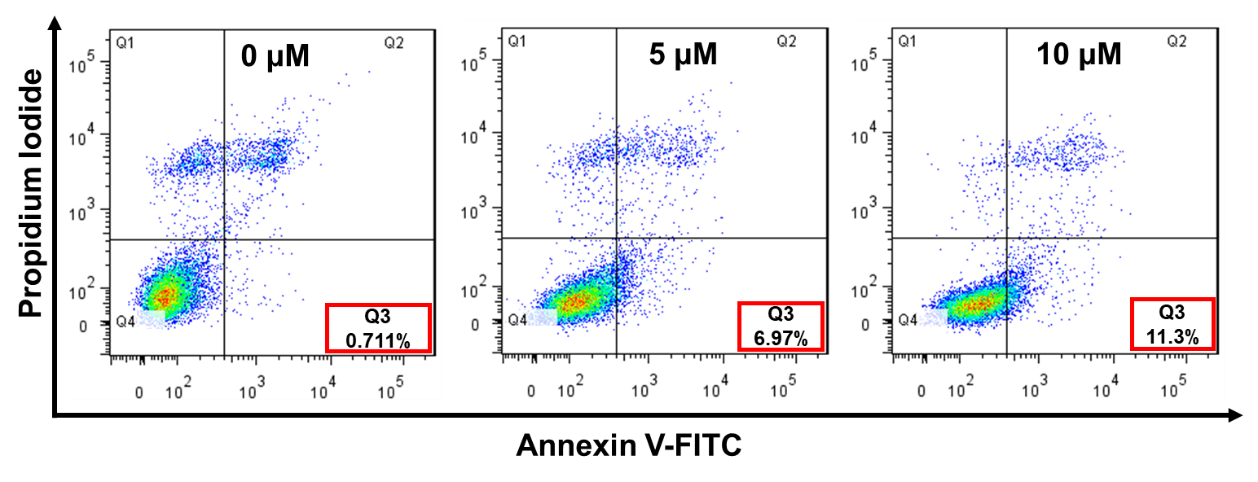
**

**Figure S20.** Detection of apoptosis in A549 cancer cells treated with **Ru-rhein** (0, 5 and 10 μM) by dual staining with Annexin V-FITC and propidium iodide. The analyzed data indicated that **Ru-rhein** showed small amount increased populations of early apoptotic cells.


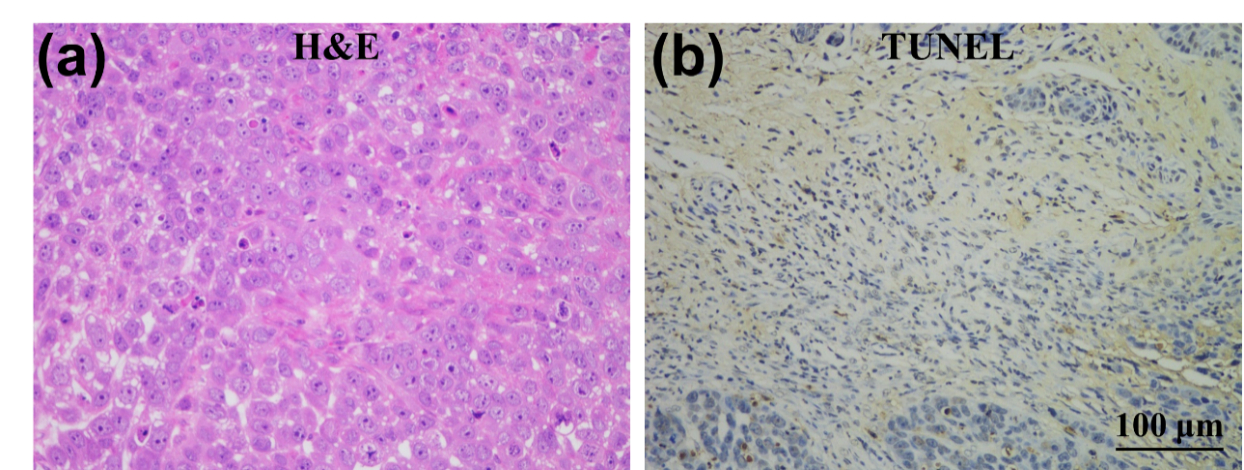


**Figure S21.** The nude mice bearing A549 tumors were intratumorally injected with the chemical synthesized **Ru-rhein** at a dosage of 8.0 mg/kg body weight, and then cell death in the tumor tissues after treatment with H&E **(a)** and TUNEL **(b)** staining assays. Scale bars are 100 μm.

**
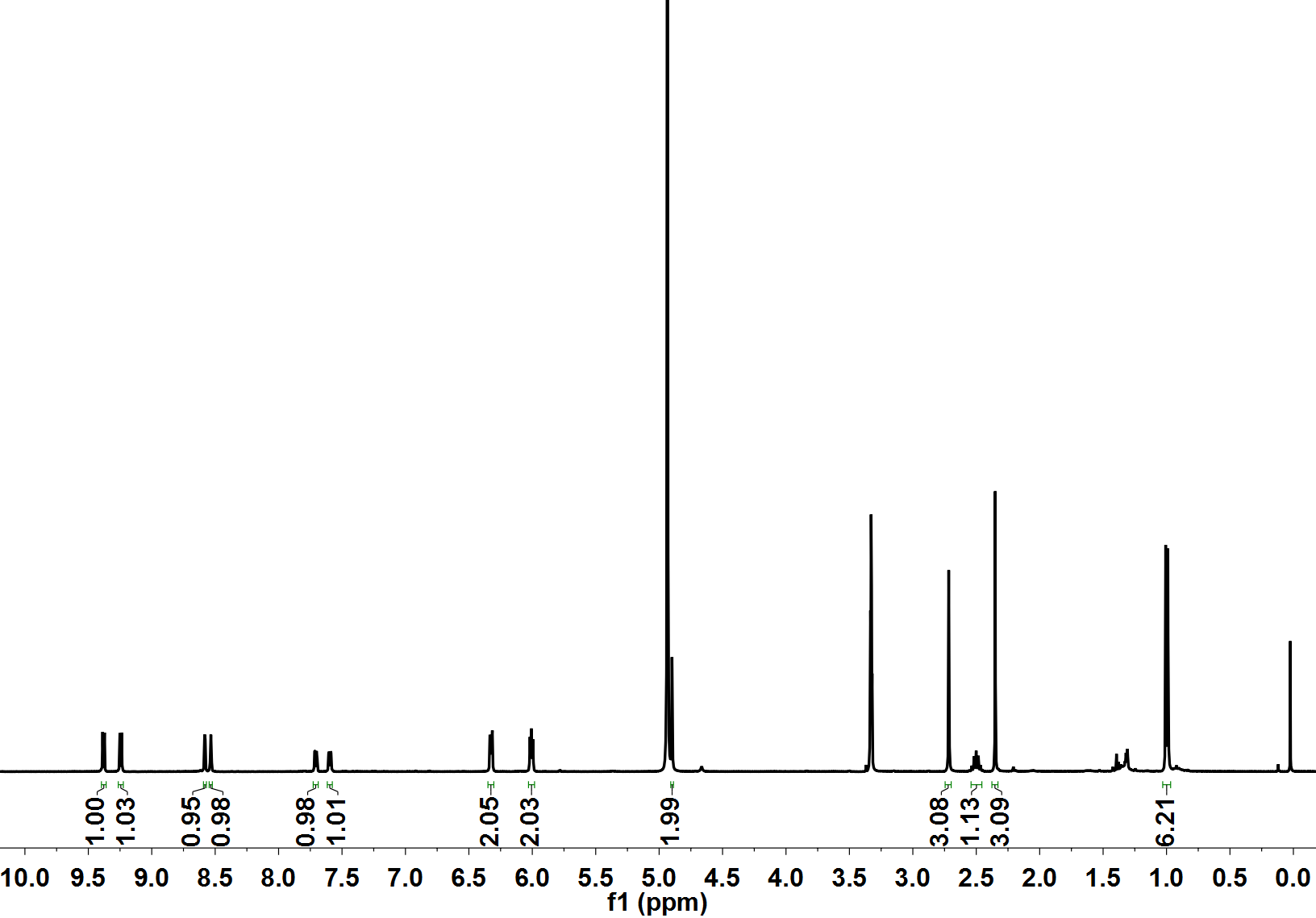
**

**Figure S22.** ^1^H NMR Data of **Os-N_3_** in CD_3_OD.

**
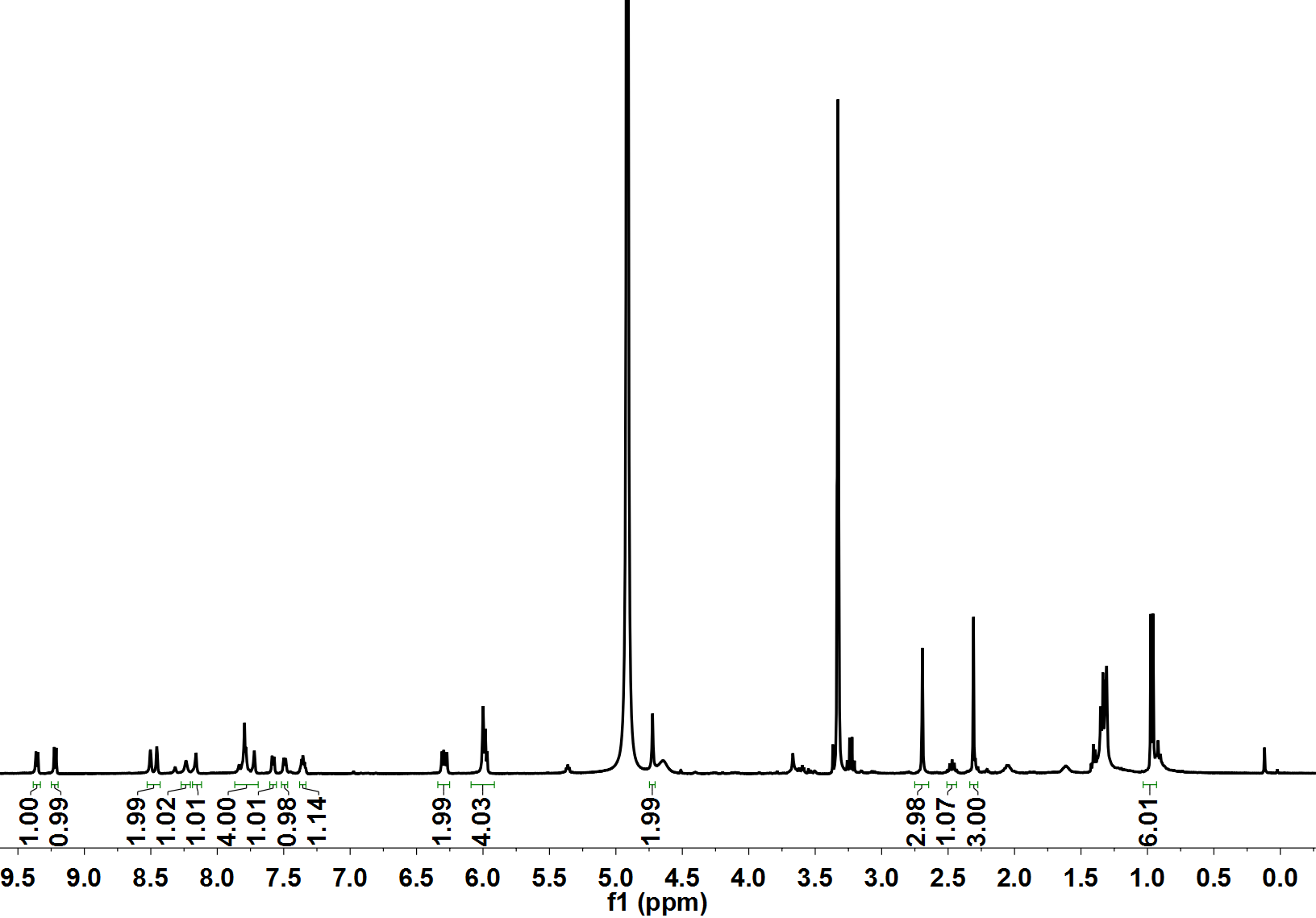
**

**Figure S23.** ^1^H NMR Data of **Os-rhein** in CD_3_OD.

**
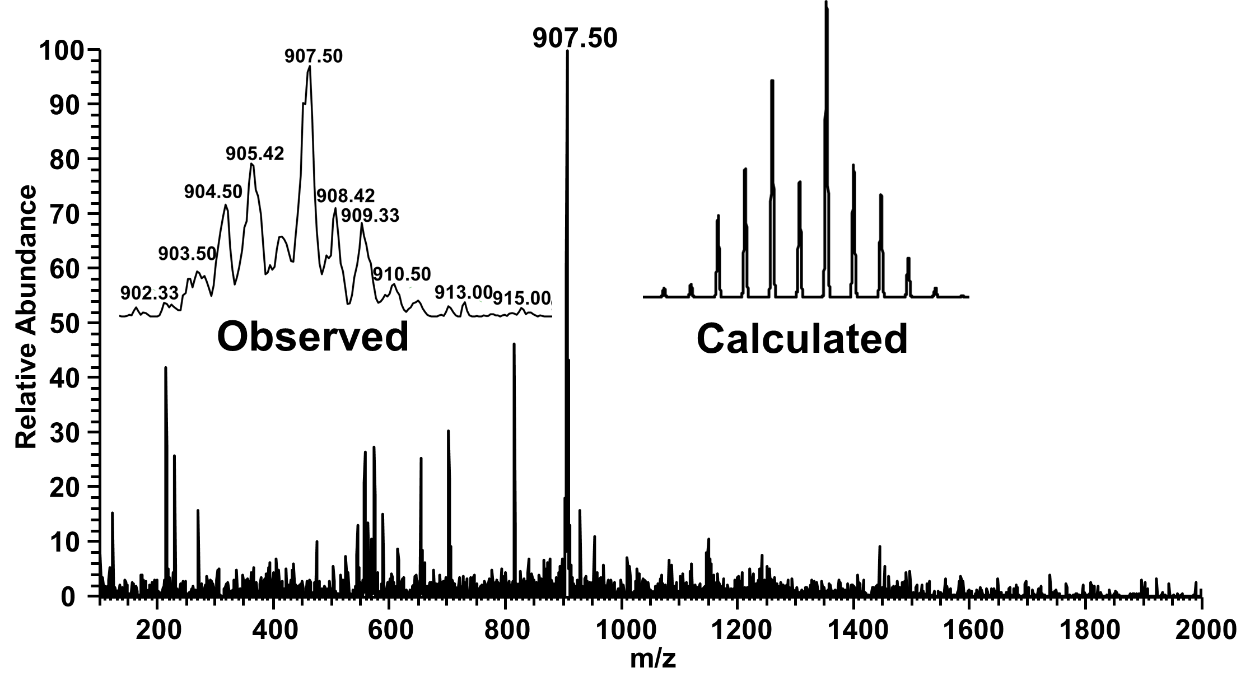
**

**Figure S24.** The ESI-MS data of **Os-rhein** in CH_3_OH solution. The observation of the m/Z peak 907.50 was consistent with the calculated results [**Os-rhein** - PF_6_]^+^ (907.21)

**Figure S25.** ^1^H NMR Data of **Ir(Cp*)-N_3_** in d_6_-DMSO.

**
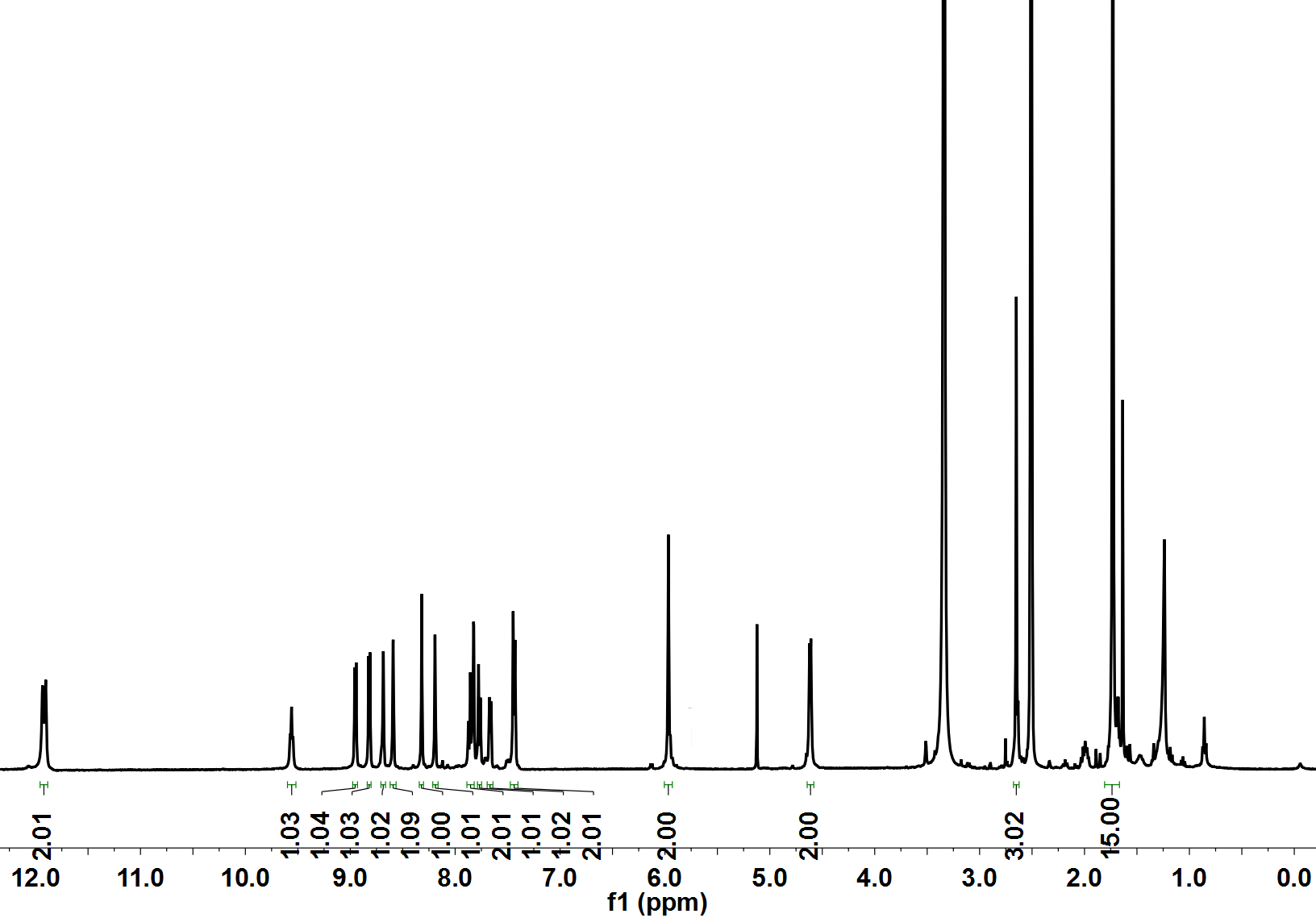
**

**Figure S26.** ^1^H NMR Data of **Ir(Cp*)-rhein** in d_6_-DMSO.

**
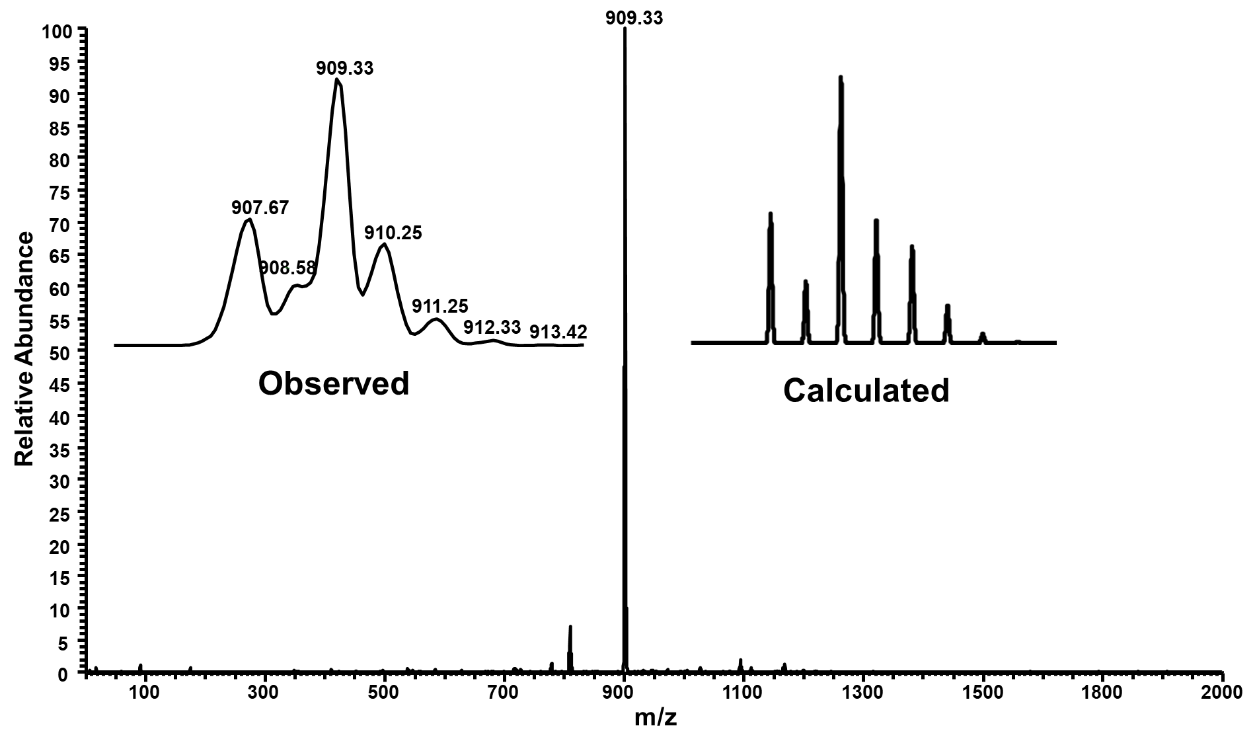
**

**Figure S27.** The ESI-MS data of **Ir(Cp*)-rhein** in CH_3_OH solution. The observation of the m/Z peak 1001.33 was consistent with the calculated results [**Ir(Cp*)-rhein -** PF_6_]^+^ (909.21).

**Table S2.** The cytotoxicities of a series of metal-arene complexes towards different cell lines based on bioorthogonally catalyzed lethality (BCL) strategy.

| Compound | | IC_50_ values (μM) for 48 h | | |
| --- | --- | --- | --- | --- |
|  |  | A549 | A2780 | MCF-7 |
| **rhein-alkyne** | | 51.2±0.6 | 46.2±1.4 | 60.7±2.1 |
| **Os-rhein** | **Os-N_3_** | >100 | >100 | >100 |
|  | **Os-rhein** | 13.3±0.6 | 15.4±0.7 | 21.5±0.8 |
| **Ir(Cp*)-rhein** | **Ir(Cp*)-N_3_** | >100 | >100 | >100 |
|  | **Ir(Cp*)-rhein** | 35.7±2.5 | 28.1±1.9 | 30.4±1.1 |

Cp*: η^5^-pentamethylcyclopentadienyl group.

**M-rhein** (M=Os, Ir) represented the product between **rhein-alkyne** and **M-N_3_** (molar ratio of 1:1) through *in situ* CuAAC in cancer cells.

**References**

[1] Xun Z, Yu T and Zeng Y *et al*. Artificial photosynthesis dendrimers integrating light-harvesting, electron delivery and hydrogen production. *J Mater Chem A* 2015; **3**: 12965-71.
